# Supplementary material for: Effect of a national infection control programme in Sweden on prosthetic joint infection incidence following primary total hip arthroplasty: a cohort study
Source: BMJ Open. 2024 Apr 29;14(4):e076576. doi: 10.1136/bmjopen-2023-076576 (PMC11086449; doi:10.1136/bmjopen-2023-076576)
Supplement: Supplementary data [file bmjopen-2023-076576supp001.pdf]

Supplemental Table 1. Diagnostic criteria walkthrough for patients treated for PJI pre PRISS (2005-08) post PRISS (2012-14); diagnostic criteria were verified sequentially.

Diagnostic criteria	(n=442)	(n=580)
1. Sinus tract communicating with the prosthesis	14 (64)	29 (168)
2. ≥2 positive periprosthetic cultures with same pathogen	59 (262)	53 (310)
3a + 3b. Presence of purulence in joint + elevated CRP/SR	5 (23)	5 (30)
3a + 3c. Presence of purulence + isolation of one periprosthetic pathogen in tissue or fluid	4 (19)	5 (31)
3b + 3c. Elevated CRP/SR + isolation of one periprosthetic pathogen in tissue or fluid	7 (29)	2 (10)
Clinical diagnosis or lack of information in questionnaires	10 (45)	5 (31)

Data presented as % (n)

CRP, C-reactive protein; SR, sedimentation rate.

Supplemental Table 2. Characteristics of patients with and without prosthetic joint infection (PJI) within 2 years of primary total hip placement surgery, 2012–2014.

	**PJI** (*n* = 580)	**Control** (*n* = 49,366)	***p*–value**
Mean age (SD)	71 (10)	69 (10)	<0.001
Female, sex	41 (240)	58 (28,405)	<0.001
BMI ≥ 30	33 (178)	24 (11,049)	<0.001
ASA classification ≥ 3	35 (200)	19 (9395)	<0.001
Indication for operation			<0.001
Primary OA	70 (401)	80 (39,231)	
Secondary OA	4 (22)	2 (1161)	
Acute trauma	12 (70)	9 (4255)	
Complication trauma	7 (40)	3 (1675)	
Sequelae of childhood hip disease	1 (7)	2 (929)	
Femoral head necrosis	3 (18)	2 (1144)	
Inflammatory joint disease	1 (13)	1 (547)	
Tumor	1 (6)	1 (285)	
Surgical approach			0.37
Direct lateral	51 (295)	48 (23,541)	
Posterior	48 (280)	51 (25,374)	
Trochanteric Osteotomy	0 (0)	0 (70)	
MIS[1]	1 (4)	1 (365)	
Implant fixation			0.22
Cemented	69 (402)	66 (32,530)	
Hybrid/reversed-hybrid	14 (80)	19 (9138)	
Uncemented	17 (98)	19 (9138)	

Data shown as % (*n*) unless otherwise indicated.

BMI, body mass index; ASA American Society of Anesthesiology class, OA, osteoarthritis; PJI, prosthetic joint infection; MIS, minimally invasive surgery; SD, standard deviation.

[1] MIS anterior, MIS posterior, Watson-Jones.

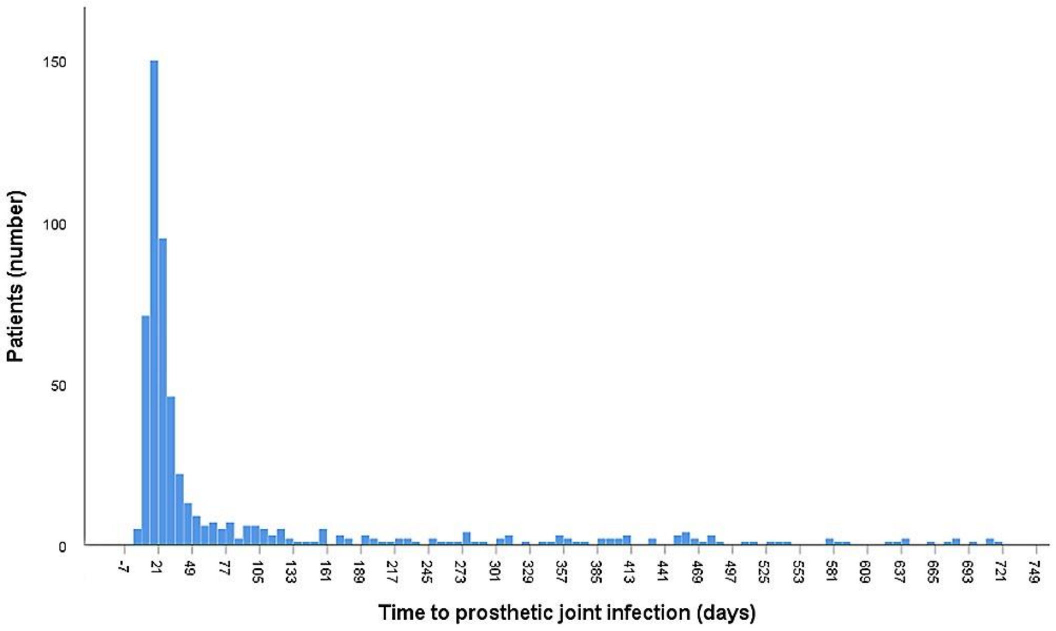

Supplemental Figure 1. Time from total hip arthroplasty to prosthetic joint infection diagnosis following the PRISS project, 2012–2014. Each bar represents 1 week.
