# Supplementary material for: Effect of a national infection control programme in Sweden on prosthetic joint infection incidence following primary total hip arthroplasty: a cohort study
Source: BMJ Open. 2024 Apr 29;14(4):e076576. doi: 10.1136/bmjopen-2023-076576 (PMC11086449; doi:10.1136/bmjopen-2023-076576)
Supplement: Author's manuscript [file bmjopen-2023-076576.draft_revisions.pdf]

BMJ Open

BMJ Open is committed to open peer review. As part of this commitment we make the peer review history of every article we publish publicly available.

When an article is published we post the peer reviewers' comments and the authors' responses online. We also post the versions of the paper that were used during peer review. These are the versions that the peer review comments apply to.

The versions of the paper that follow are the versions that were submitted during the peer review process. They are not the versions of record or the final published versions. They should not be cited or distributed as the published version of this manuscript.

BMJ Open is an open access journal and the full, final, typeset and author-corrected version of record of the manuscript is available on our site with no access controls, subscription charges or pay-per-view fees (http://bmjopen.bmj.com).

If you have any questions on BMJ Open's open peer review process please email info.bmjopen@bmj.com

BMJ Open

The effect of a national infection control program in Sweden on prosthetic joint infection incidence following primary total hip arthroplasty.

Journal:	*BMJ Open*
Manuscript ID	bmjopen-2023-076576
Article Type:	Original research
Date Submitted by the Author:	15-Jun-2023
Complete List of Authors:	Wildeman, Peter; Orebro universitet, Department of Orthopedics, Faculty of Medicine and Health<br>Rolfson, Ola; University of Gothenburg, Department of Orthopaedics, Institute of Clinical Sciences, Sahlgrenska Academy; Registercentrum Vastra Gotaland, Swedish Arthroplasty Register<br>Wretenberg, Per; Örebro University, Department of Orthopedics, Faculty of Medicine and Health<br>Nåtman, Jonatan; Centre of Registers Vastra Gotaland, Swedish Arthroplasty Register<br>Gordon, Max; Karolinska Institute, Department of Clinical Sciences, Danderyd Hospital<br>Söderqvist, Bo; Örebro University, Department of Infectious Diseases, Faculty of Medicine and Health; Örebro University, School of Medical Sciences, Faculty of Medicine and Health<br>Lindgren, Viktor; Karolinska Institutet, Department of Molecular Medicine and Surgery
Keywords:	Hip < ORTHOPAEDIC & TRAUMA SURGERY, Infection control < INFECTIOUS DISEASES, Primary Prevention, Epidemiology < INFECTIOUS DISEASES

SCHOLARONE™
Manuscripts

I, the Submitting Author has the right to grant and does grant on behalf of all authors of the Work (as defined in the below author licence), an exclusive licence and/or a non-exclusive licence for contributions from authors who are: i) UK Crown employees; ii) where BMJ has agreed a CC-BY licence shall apply, and/or iii) in accordance with the terms applicable for US Federal Government officers or employees acting as part of their official duties; on a worldwide, perpetual, irrevocable, royalty-free basis to BMJ Publishing Group Ltd ("BMJ") its licensees and where the relevant Journal is co-owned by BMJ to the co-owners of the Journal, to publish the Work in this journal and any other BMJ products and to exploit all rights, as set out in our licence.

The Submitting Author accepts and understands that any supply made under these terms is made by BMJ to the Submitting Author unless you are acting as an employee on behalf of your employer or a postgraduate student of an affiliated institution which is paying any applicable article publishing charge ("APC") for Open Access articles. Where the Submitting Author wishes to make the Work available on an Open Access basis (and intends to pay the relevant APC), the terms of reuse of such Open Access shall be governed by a Creative Commons licence – details of these licences and which Creative Commons licence will apply to this Work are set out in our licence referred to above.

Other than as permitted in any relevant BMJ Author's Self Archiving Policies, I confirm this Work has not been accepted for publication elsewhere, is not being considered for publication elsewhere and does not duplicate material already published. I confirm all authors consent to publication of this Work and authorise the granting of this licence.

For peer review only

The effect of a national infection control program in Sweden on prosthetic joint infection incidence following primary total hip arthroplasty.

Peter Wildeman [1,2], Ola Rolfson [3,4], Per Wretenberg [1,2], Jonatan Nåtman[4], Max Gordon [5], Bo Söderquist [2,6], and Viktor Lindgren[7]

1 Department of Orthopedics, Faculty of Medicine and Health, Örebro University, Örebro

2 School of Medical Sciences, Faculty of Medicine and Health, Örebro University, Örebro

3 Department of Orthopedics, Institute of Clinical Sciences, Sahlgrenska Academy, University of Gothenburg, Gothenburg

4 Swedish Arthroplasty Register, Centre of Registers, Västra Götalandsregionen, Gothenburg

5 Department of Clinical Sciences, Karolinska Institutet, Danderyd Hospital, Stockholm

6 Department of Infectious Diseases, Faculty of Medicine and Health, Örebro University, Örebro

7 Department of Molecular Medicine and Surgery, Karolinska Institutet, Stockholm, Sweden

Word count: Abstract 245

Manuscript, excluding abstract, 2859

Corresponding author:

Peter Wildeman

Department of Orthopedics

Örebro University Hospital

SE-70185 Örebro

Sweden

For peer review only

24 Telephone: +46196021000

25 peter.wildeman@regionorebrolan.se

For peer review only

For peer review only - http://bmjopen.bmj.com/site/about/guidelines.xhtml

Abstract

Objectives

Prosthetic joint infection (PJI) is a serious complication following total hip arthroplasty (THA) entailing increased mortality, decreased quality of life, and high healthcare costs.

The primary aim was to investigate whether the national project: Prosthesis Joint Infections shall be stopped (PRISS) reduced PJI incidence after primary THA; the secondary aim was to evaluate other possible benefits of PRISS, such as shorter time to diagnosis.

Design

Retrospective cohort study.

Setting

In 2009 a nationwide, multidisciplinary infection control program was launched in Sweden, PRISS, which aimed to reduce the PJI burden by 50%.

Participants

We obtained data on patients undergoing primary THA in Sweden (n = 45,723 patients, 49,946 THAs), 2012–2014. Using personal identity numbers, this cohort was matched with the Swedish Prescribed Drug Registry. Medical records of patients with ≥4 weeks antibiotic consumption were reviewed to verify PJI diagnosis (n = 2240, 2569 THAs).

Results

The cumulative incidence of PJI following the PRISS project was 1.2% [95% CI 1.1-1.3] as compared to 0.9% [95% CI 0.8-1.0] before. Cox regression models for the PJI incidence post PRISS indicates there were no statistical significance difference versus pre PRISS (HR 1.1 [95% CI 0.9-1.3]. There were similar time to PJI diagnosis after the PRISS project 24 vs 23 days (p=0.5).

For peer review only - http://bmjopen.bmj.com/site/about/guidelines.xhtml

Conclusions

Despite the comprehensive nationwide PRISS project, Swedish PJI incidence was higher after the project and time to diagnosis remained unchanged. Factors contributing to PJI, such as increasing obesity, higher ASA class, and more fractures as indications, explain the PJI increase among primary THA patients.

Strengths and limitations of this study

- This is the first study of a national program aimed to reduce the burden of prosthetic joint infections (PJIs) of the hip.
- Utilized the exact same method for data capturing as a previous study investigating the PJI incidence in Sweden.
- Long follow-up time to capture PJI events.
- Very high response rate to questionnaire from all units in Sweden operating total hip arthroplasty.

Keywords: PJI, Prosthetic Joint Infection, Prevention, Arthroplasty, Hip.

Introduction

In a world with an increasing elderly population (1) whose members wish to live healthy and mobile lives for most of their lifespans, artificial hip joints improve quality of life (QoL) for millions of patients every year (2). Excellent long-term pain reduction and improved mobility have popularized the surgical procedure of total hip arthroplasty (THA). However, as with any surgical intervention, THA is associated with procedure-related complications. Aseptic loosening, repeated hip dislocation, thromboembolic event, and prosthetic joint infection (PJI) are the most common complications (3-5). PJIs have multifactorial causes, and extensive research has explored the risk factors for infection (3). Those associated with PJIs are both

For peer review only

endogenous factors such as male gender, obesity, poor diabetic control, and active smoking (6, 7), and exogenous factors such as timing of prophylactic antibiotics, surgical site preparation, and laminar airflow (8-10). PJI as reason for reoperation has gradually increased in recent decades and is now the most common indication for reoperation within two years of primary THA (4). PJIs are associated with increased mortality (11-13), lower QoL (13, 14), and higher costs for healthcare providers (15, 16). In an attempt to reduce the PJI burden, the Swedish Orthopedic Association and Swedish National Patient Insurance started 2009 a nationwide, multidisciplinary infection control program: PRISS (Prosthetic Related Infections Shall be Stopped). All orthopedic surgical units (n = 81) in Sweden participated in the PRISS project and were reviewed. The goal of the project was to reduce PJI incidence following total hip and knee arthroplasty in Sweden by 50%. A study by Lindgren et al (17) estimated the PJI incidence to 0.9% after primary THA, the years prior to PRISS, 2005-2008. The effect of the PRISS project on PJI after THA is however unknown. The primary aim of the present study was therefore to investigate if the PRISS project reduced the incidence of PJI following THA; the secondary aims were to evaluate other possible benefits of the initiative, i.e., shorter time to diagnosis, and to identify pathogens involved in PJIs.

Methods and analysis

Design

The PRISS study is a retrospective cohort study.

Registries

All public and private orthopedic clinics performing THA operations report to the Swedish Arthroplasty Register (SAR). Approximately 98% of all THAs in Sweden are reported to the registry (4). Details such as indication for surgery, type of implant, surgical approach, body mass index (BMI), and American Society of Anesthesiology class (ASA) are reported. The

For peer review only - http://bmjopen.bmj.com/site/about/guidelines.xhtml

Swedish Prescribed Drug Registry (SPDR) is operated by the Swedish National Board of Health and Welfare, and all pharmacies in Sweden automatically report complete details regarding dispensed drugs, including type of drug, amount dispensed, instructions from prescribing doctor, and dates of prescription and dispensing.

Swedish National Patient Insurance and the PRISS project

The Swedish National Patient Insurance (SNPI) is a public insurance company jointly owned by its policy holders, the Swedish regions and county councils. Its primary goals are to insure its owners, handle claims, compensate patients affected by health-care-associated complications, and address patient safety. In 2008, 33% of all claims were from the orthopedic specialty (18). In 2009,the PRISS project was initiated jointly by SNPI and the Swedish Orthopedic Association (18). The project started in 2009 and by 2012 all orthopedic units (*n* = 81) had been enrolled in it. It was an interdisciplinary project for safer THA and TKA operations involving several professional organizations such as the Swedish associations for orthopedic, infectious disease specialists, surgical nursing, orthopedic nursing, physical therapy, and infection control. A self-assessment tool, site inspections, and expert group evaluations were performed on all units to evaluate the routines before, during and after arthroplasty surgery (18). Eighty-five percent of all implemented measures were improvement in procedures and protocols. Factors such as optimizing patients before surgery, basic hygiene routines, optimizing antibiotic prophylaxis, improving operating theatres (e.g. CFU measurements, locked rooms and maximum number of persons in operating rooms), and postoperative wound care were among those factors addressed (Table 1). However, the impact of optimization of these measures in the setting of the PRISS project, on the PJI risk, has not been explored.

For peer review only - http://bmjopen.bmj.com/site/about/guidelines.xhtml

Study design, participants, and setting

A new method of postoperative infection surveillance introduced by Lindgren et al. combined national medical registry data with medical record review (17), determining PJI cumulative incidence within 2 years of primary THA to be 0.9% (95% CI 0.9-1.0) between 2005 and 2008. From that study, we extracted patient demographic data, on patients with a primary THA and a PJI diagnosis to compare with the post PRISS cohort (Table 2). The present study used the same method, including diagnostic criteria for PJI (19), to determine the incidence of PJI within 2 years of primary THA over the 2012–2014 period. From SAR we extracted all primary THA procedures (n = 49,946 in 45,723 patients) between 1 January 2012 and 31 December 2014 (Figure 1). Using personal identity numbers, we matched this cohort with the SPDR and identified 2240 patients undergoing a total of 2569 primary THAs with continuous outpatient consumption of antibiotics for a minimum of 4 weeks minimum of 2 years after each primary THA. For each THA, a questionnaire was sent to the operating unit, where a physician reviewed the medical records of that patient to verify that he/she had been treated for PJI. From the questionnaire, information on date of diagnosis, clinical presentation, and surgical treatment was obtained (Supplemental Table 1). Ninety-nine percent (n = 2531/2569) of the questionnaires were returned along with the microbiology results. PJI after revision THA as well as superficial infections were excluded.

Statistics

Continuous variables were presented as means (with standard deviations) and medians (with inter-quantile ranges), as appropriate. The χ^2 test and Fisher's exact test were used to compare categorical data such as sex, BMI ≥ 30, and ASA ≥ 3. The t-test was used to evaluate between-group differences in age. Cumulative incidence of PJI with 95 % confidence intervals were estimated using the Kaplan-Meier estimator. Hazard ratios with 95 %

For peer review only - http://bmjopen.bmj.com/site/about/guidelines.xhtml

confidence intervals were estimated using Cox regression adjusted for age, sex, and diagnosis (OA/other). Patients were followed for two years from primary operation until the first PJI and censored for death and revision for other causes than PJI. A sensitivity analysis, in the part of the cohort with complete data, a Cox regression also adjusted for ASA and BMI were estimated.

All tests were two-tailed, and statistical significance was defined as a *p*-value < 0.05 or a 95% confidence interval (CI)) excluding an odds ratio (OR) equal to 1.00. Statistical analyses were performed using SPSS version 25 (IBM Corp., Armonk, NY, USA).

Results

Five hundred and eighty patients treated for a PJI were identified (Supplemental Table 2), resulting in a nationwide 2-year cumulative incidence of 1.2% [95% CI 1.1-1.3] over the 2012–2014 period as compared to 0.9% [95% CI 0.8-1.0] in the 2005-2008 period. There was an increasing trend of PJI incidence during the study period (Figure 2) and a total increase of 30% during the whole study period. A Cox regression model indicated no statistically significant difference in the cumulative incidence pre- versus post-PRISS PJI (HR 1.1 [95% CI 0.9–1.3],).

The highest incidence was noted in university hospitals (1.5%), followed by regional (1.4%) and county and private hospitals (both 0.9%). After controlling for the potential confounders of age, sex, indication for surgery, BMI ≥ 30, and ASA ≥ 3, we found no statistically significant differences in PJI incidence among the different hospital classes. For individual orthopedic units, the cumulative incidence ranged from 0.0 to 5.9%; in two units the incidence had decreased and in three it had increased compared with before the PRISS project. The median time from primary THA to PJI diagnosis was 23 days (IQR 48) (Supplemental figure 1), not significantly different from the 24 days before the PRISS project (IQR 46, *p*=0.51).

For peer review only - http://bmjopen.bmj.com/site/about/guidelines.xhtml

169

170 In Table 3 the distribution of the microbial agents isolated from PJIs pre and post PRISS is
171 presented. The most common microorganisms, isolated in pure culture or as part of
172 polymicrobial growth, were *Staphylococcus* spp. (72% pre vs 68% post, *p*=0.9). In pure
173 culture, *Staphylococcus aureus*, was the most abundant species, with a minor increase post
174 PRISS (20% vs 26%, *p*=0.04), and one isolate was found to be methicillin resistant (i.e.,
175 MRSA) vs. none pre PRISS. Coagulase-negative staphylococci (CoNS) were the second most
176 commonly isolated group of bacteria (21% vs 24%, *p*=0.2). The number of polymicrobial
177 PJIs, with 2–4 different bacterial species, did not differ significantly pre vs post PRISS (27%
178 vs 25%, *p*=0.5).

179 Discussion

180 We investigated the effect of PRISS on PJI incidence in patients with primary THA in the
181 years following the project (i.e., 2012–2014) with a follow-up period of 2 years. We also
182 examined time to diagnosis and infecting microorganisms. We found a 30 % increase in PJI
183 incidence compared with before the PRISS project, i.e. 1.2 vs. 0.9% (17), but there was no
184 statistically significant difference, after controlling for confounders such as BMI, ASA, and
185 indication for surgery. Time to diagnosis remained unchanged pre vs. post PRISS (i.e.,
186 median 23 vs. 24 days).

187

188 Different sources of error may have affected the PJI incidence level reported here. One source
189 of error was that the specific reason for antibiotic prescription could not be established in all
190 cases. For example, if a patient had moved from the county where s/he initially underwent
191 surgery, local medical record review was impossible. Another limitation concerns identifying
192 the patient cohort through SPDR. Since SPDR only handles outpatient prescriptions, patients
193 who died in hospital or were hospitalized during the course of treatment could not be

For peer review only - http://bmjopen.bmj.com/site/about/guidelines.xhtml

detected; however, the mortality rate is very low following arthroplasty (20), limiting this effect. According to the Swedish national guidelines (21), the advocated antibiotic treatment for PJI is 2 weeks of intravenous and 10 weeks of oral antibiotic therapy. Inpatient care for such long time is rare in Sweden which minimizes the immortal time bias. In addition, the sensitivity of the 2011 MSIS criteria has been questioned (22). All these limitations may serve to underestimate the true incidence of PJI. Nevertheless, the study was performed using the exact same methodology as in the prior study by Lindgren et al, (17) establishing the PJI incidence before PRISS, and having the same limitations in both studies makes the results comparable. SAR started to collect BMI and ASA data in 2008, limiting the number of patients for whom this information is available. Over the study period we could identify a trend of increasing comorbidity of the patients (age, BMI, ASA class) and proportion of patients with hip fracture/secondary OA/sequelae after fracture.

As the cause of PJI is multifactorial any initiative to reduce the infection incidence must be broad and include all health care disciplines involved. The PRISS project included implementations of a bundle of actions to improve pre-, intra- and post-operative care following expert reviews(18). All recommendations were voluntary for the orthopaedic units, possibly limiting the effect of best-practice methods. It is also possible that there are unknown confounders contribution to an increase in PJI incidence that were not measured.

Most infections were diagnosed in the first two weeks following THA and infections were only sporadically identified after the first 90 days (Supplemental figure 3). This implies that most infections originate from the surgical procedure or during the early postoperative period, indicating that properly timing the first dose of prophylactic antibiotics as well as very careful wound care in the early postoperative period are essential. This also implies that the improved

For peer review only

For peer review only - http://bmjopen.bmj.com/site/about/guidelines.xhtml

post-operative procedures and protocols of PRISS, such as wound treatment protocols, phone screening, and patient information, had limited impact on PJI incidence. There was no change in time to diagnosis compared with before the PRISS project (i.e., 23 vs. 24 days). This time to diagnosis is likely acceptable, as infection treatment has a chance of successful outcome (i.e., infection eradication) if initiated within 28 days of infection for acute infections (21, 23-25).

The risk of PJI following the first year of surgery is approximately 1% annually (3, 17), a risk that seemed to be unaffected by the PRISS project. Late infections include both those contracted during surgery or the early postoperative phase progressing to chronic PJIs, and acute hematogenous infections, which by definition could not be affected by the PRISS project measures. Although hematogenous infections can be treated successfully with a debridement, antibiotics, and implant retention (DAIR) procedure within 3 weeks of infection onset (25), this treatment algorithm is less likely to be successful in a procedure for chronic PJI (24, 26, 27). In this study we have not tried to distinguish between the two types of late infections.

The PJI incidence before PRISS was lower at the beginning of the study period, and there was a trend of increasing incidence at the end of 2008(17). A trend of increasing PJI incidence could also be detected over the 2012–2014 period, representing a continuation of the increasing incidence noted later in the 2007–2008 period (Figure 2). This rising trend could have been because determination to achieve improvement diminished following the end of PRISS. The PRISS project was a national intervention with broad support from participants from all professional organizations in collaboration with SNPI and all orthopedic units. Despite broad support and an evidence-based approach, PRISS project did not reduce PJI

For peer review only - http://bmjopen.bmj.com/site/about/guidelines.xhtml

incidence. During the 2008–2014 period, the number of patients with severe systemic disease (ASA ≥ 3) increased, possibly contributing to the increasing PJI incidence over this period(28). More obese patients and patients with hip fractures underwent THA post PRISS (Table 2). Early operation is very important for a patient with a hip fracture (29) , but may also risk non-compliance to preoperative routines, thus contributing to the PJI risk. Thompson et al concluded that the PRISS project did not had any effect on PJI incidence after primary total knee arthroplasty (TKA) with similar pre and post PJI incidence(30), but did not explore the effect of co-morbidities . If the trend of increasing co-morbidities apply to the patients with TKA, this could imply that PRISS project could have had an effect on the PJI incidence for TKA (30) . However, fracture as indication for primary TKA is rare as compared to THA, limiting this effect on PJI incidence for TKA. Although PJI incidence has progressively increased over the years in Sweden, it is comparable to that reported in an international context (31, 32). Some risk factors for infection cannot be influenced (e.g., sex, age, and reason for THA), whereas some, although modifiable (e.g., heart failure, diabetes, and obesity), are hard to influence especially if the patient is in pain and has difficulty walking before THA, meaning that they were largely unaffected by the preoperative measures of the PRISS initiative. However, it is possible that the PJI incidence might have been even higher without the PRISS project and its various benefits, such as more patients receiving correct initial treatment and therefore being cured of infection (3, 30).

Staphylococci were by far the most common etiological agent of PJI, in agreement with international reports (33) and national observations before PRISS (17, 34). Since only one methicillin-resistant *S. aureus* (MRSA) case was found, the vast majority of *S. aureus* were susceptible to most antibiotics. However, on the contrary, many of the CoNS were not determined to species level, and *S. epidermidis*, the most common staphylococcal species

For peer review only - http://bmjopen.bmj.com/site/about/guidelines.xhtml

among CoNS causing PJI, are in contrast to *S. aureus* often multidrug resistant (35). Whether a pattern of increasing multidrug resistance could explain the increasing PJI incidence was not evaluated here.

Conclusions

The PRISS project did not reduce the PJI incidence and the time to diagnosis remained unchanged. This study indicates a lack of impact of non-mandatory recommended measures, despite being evidence based, to be implemented to reduce multifactorial complications such as PJI of the hip when the incidence is low. Higher proportions of obese, comorbid, and fracture patients likely explain the minor increase in PJI incidence, and it is possible that in modern health care, endogenous risk factors might affect the PJI incidence more than exogenous (and modifiable) risk factors. Further studies of patients with a high risk of PJI, such as fracture, revision surgery and comorbid patients, are warranted to reduce overall incidence of PJI and its severe complications. An increased focus on PJIs may have contributed to a better treatment outcome, but this needs further studies.

Ethical approval

This study was approved by the Regional Ethical Review Board of Gothenburg, Sweden (reference number 2017/329-17).

Conflicts of interests

The authors declare they have no conflicts of interests.

For peer review only - http://bmjopen.bmj.com/site/about/guidelines.xhtml

Author contributions

All authors designed the study. VL had project supervision. PW, MG and JN analyzed the data. PW wrote the manuscript with contributions from OR, PWR, MG, JN, BS, and VL. All authors approved the final version of the manuscript.

Acknowledgments

The authors would like to thank all orthopedic units and SAR contact physicians for review of the medical records. The authors would also like to thank Ann-Britt Wall, Department of Orthopedics Region Örebro County, for administrative support.

Funding

The authors would like to thank the funding organizations, Research Committee of Örebro University, Region Örebro County, Sweden and Patientförsäkringen LÖF, Sweden, for financial support of the study. The funders had no role in study design, data collection and analysis, decision to publish or preparation of the manuscript.

For peer review only - http://bmjopen.bmj.com/site/about/guidelines.xhtml

References

1. Ritchie H, Roser M. Age Structure. 2019 [cited 2021 04/20]; Available from: https://ourworldindata.org/age-structure.
2. Learmonth ID, Young C, Rorabeck C. The operation of the century: total hip replacement. Lancet. 2007 Oct 27;370(9597):1508-19.
3. Tande AJ, Patel R. Prosthetic joint infection. Clin Microbiol Rev. 2014 Apr;27(2):302-45.
4. Kärrholm J, Rogmark C, Nauclér E, Vinblad J, Mohaddes M, Rolfson O. Swedish Hip Arthroplasty Register Annual Report 2018. . 2018;2018(1st January).
5. Healy WL, Iorio R, Clair AJ, Pellegrini VD, Della Valle CJ, Berend KR. Complications of Total Hip Arthroplasty: Standardized List, Definitions, and Stratification Developed by The Hip Society. Clin Orthop Relat Res. 2016 Feb;474(2):357-64.
6. Iannotti F, Prati P, Fidanza A, Iorio R, Ferretti A, Pèrez Prieto D, et al. Prevention of Periprosthetic Joint Infection (PJI): A Clinical Practice Protocol in High-Risk Patients. Trop Med Infect Dis. 2020 Dec 11;5(4).
7. Sørensen LT. Wound healing and infection in surgery. The clinical impact of smoking and smoking cessation: a systematic review and meta-analysis. Arch Surg. 2012 Apr;147(4):373-83.

For peer review only - http://bmjopen.bmj.com/site/about/guidelines.xhtml

8. Alamanda VK, Springer BD. The prevention of infection: 12 modifiable risk factors. Bone Joint J. 2019 Jan;101-b(1_Supple_A):3-9.

9. Lenguerrand E, Whitehouse MR, Beswick AD, Kunutsor SK, Burston B, Porter M, et al. Risk factors associated with revision for prosthetic joint infection after hip replacement: a prospective observational cohort study. Lancet Infect Dis. 2018 Sep;18(9):1004-14.

10. Dale H, Fenstad AM, Hallan G, Havelin LI, Furnes O, Overgaard S, et al. Increasing risk of prosthetic joint infection after total hip arthroplasty. Acta Orthop. 2012 Oct;83(5):449-58.

11. Natsuhara KM, Shelton TJ, Meehan JP, Lum ZC. Mortality during total hip periprosthetic joint infection. J Arthroplasty. 2019 Jul;34(7s):S337-s42.

12. Gundtoft PH, Pedersen AB, Varnum C, Overgaard S. Increased Mortality After Prosthetic Joint Infection in Primary THA. Clin Orthop Relat Res. 2017 Nov;475(11):2623-31.

13. Wildeman P, Rolfson O, Söderquist B, Wretenberg P, Lindgren V. What Are the Long-term Outcomes of Mortality, Quality of Life, and Hip Function after Prosthetic Joint Infection of the Hip? A 10-year Follow-up from Sweden. Clin Orthop Relat Res. 2021 May 31.

14. Poulsen NR, Mechlenburg I, Soballe K, Lange J. Patient-reported quality of life and hip function after 2-stage revision of

For peer review only - http://bmjopen.bmj.com/site/about/guidelines.xhtml

chronic periprosthetic hip joint infection: a cross-sectional study. Hip Int. 2018 Jul;28(4):407-14.

15. Peel TN, Cheng AC, Lorenzo YP, Kong DC, Buising KL, Choong PF. Factors influencing the cost of prosthetic joint infection treatment. J Hosp Infect. 2013 Nov;85(3):213-9.

16. Lora-Tamayo J, Senneville É, Ribera A, Bernard L, Dupon M, Zeller V, et al. The Not-So-Good Prognosis of Streptococcal Periprosthetic Joint Infection Managed by Implant Retention: The Results of a Large Multicenter Study. Clin Infect Dis. 2017 Jun 15;64(12):1742-52.

17. Lindgren V, Gordon M, Wretenberg P, Karrholm J, Garellick G. Deep infection after total hip replacement: a method for national incidence surveillance. Infect Control Hosp Epidemiol. 2014 Dec;35(12):1491-6.

18. Gustafson P, Schultz T, Stefansdottir A. PRISS – ProsthesisRelated Infections Shall be Stopped 2014.

19. Parvizi J, Zmistowski B, Berbari EF, Bauer TW, Springer BD, Della Valle CJ, et al. New definition for periprosthetic joint infection: from the Workgroup of the Musculoskeletal Infection Society. Clin Orthop Relat Res. 2011 Nov;469(11):2992-4.

20. Dale H, Børsheim S, Kristensen TB, Fenstad AM, Gjertsen JE, Hallan G, et al. Perioperative, short-, and long-term mortality related to fixation in primary total hip arthroplasty: a study on 79,557 patients in the -Norwegian Arthroplasty Register. Acta Orthop. 2020 Apr;91(2):152-8.

For peer review only - http://bmjopen.bmj.com/site/about/guidelines.xhtml

21. Swedish Society for Infectious Diseases. Vårdprogram för Led- och skelettinfektioner 2018. https://infektion.net/2018; 128]. Available from: https://infektion.net/wp-content/uploads/2018/11/2018-vardprogram-led-och-skelettinfektioner-final-2018-11-29.pdf.

22. Koh IJ, Cho WS, Choi NY, Parvizi J, Kim TK. How accurate are orthopedic surgeons in diagnosing periprosthetic joint infection after total knee arthroplasty?: A multicenter study. Knee. 2015 Jun;22(3):180-5.

23. Osmon DR, Berbari EF, Berendt AR, Lew D, Zimmerli W, Steckelberg JM, et al. Executive summary: diagnosis and management of prosthetic joint infection: clinical practice guidelines by the Infectious Diseases Society of America. Clin Infect Dis. 2013 Jan;56(1):1-10.

24. Zimmerli W. Clinical presentation and treatment of orthopaedic implant-associated infection. J Intern Med. 2014 Aug;276(2):111-9.

25. Wouthuyzen-Bakker M, Sebillotte M, Lomas J, Taylor A, Palomares EB, Murillo O, et al. Clinical outcome and risk factors for failure in late acute prosthetic joint infections treated with debridement and implant retention. J Infect. 2019 Jan;78(1):40-7.

26. Wouthuyzen-Bakker M, Sebillotte M, Lomas J, Kendrick B, Palomares EB, Murillo O, et al. Timing of implant-removal in

For peer review only - http://bmjopen.bmj.com/site/about/guidelines.xhtml

late acute periprosthetic joint infection: A multicenter observational study. J Infect. 2019 Sep;79(3):199-205.

27. Maillet M, Pavese P, Bruley D, Seigneurin A, François P. Is prosthesis retention effective for chronic infections in hip arthroplasties? A systematic literature review. Eur J Clin Microbiol Infect Dis. 2015 Aug;34(8):1495-502.

28. Kärrholm J, Lindahl H, Malchau H, Mohaddes M, Rogmark C, Rolfson O. The Swedish Hip Arthroplasty Register Annual Report 2015. 2016.

29. Simunovic N, Devereaux Pj Fau - Sprague S, Sprague S Fau - Guyatt GH, Guyatt Gh Fau - Schemitsch E, Schemitsch E Fau - Debeer J, Debeer J Fau - Bhandari M, et al. Effect of early surgery after hip fracture on mortality and complications: systematic review and meta-analysis. (1488-2329 (Electronic)).

30. Thompson O, A WD, Lindgren V, Gordon M, Robertsson O, Stefánsdóttir A. Similar periprosthetic joint infection rates after and before a national infection control program: a study of 45,438 primary total knee arthroplasties. Acta Orthop. 2022 Jan 3;93:3-10.

31. Gundtoft PH, Pedersen AB, Schønheyder HC, Møller JK, Overgaard S. One-year incidence of prosthetic joint infection in total hip arthroplasty: a cohort study with linkage of the Danish Hip Arthroplasty Register and Danish Microbiology Databases. (1522-9653 (Electronic)).

For peer review only - http://bmjopen.bmj.com/site/about/guidelines.xhtml

32. McMaster Arthroplasty Collaborative (MAC). Risk Factors for Periprosthetic Joint Infection Following Primary Total Hip Arthroplasty: A 15-Year, Population-Based Cohort Study. J Bone Joint Surg Am. 2020;2020;102(6):503-509(1535-1386 (Electronic)).

33. Flurin L, Greenwood-Quaintance KE, Patel R. Microbiology of polymicrobial prosthetic joint infection. Diagn Microbiol Infect Dis. 2019 Jul;94(3):255-9.

34. Stefánsdóttir A, Johansson D, Knutson K, Lidgren L, Robertsson O. Microbiology of the infected knee arthroplasty: report from the Swedish Knee Arthroplasty Register on 426 surgically revised cases. Scand J Infect Dis. 2009;41(11-12):831-40.

35. Månsson E, Bech Johannesen T, Nilsdotter-Augustinsson Å, Söderquist B, Stegger M. Comparative genomics of Staphylococcus epidermidis from prosthetic-joint infections and nares highlights genetic traits associated with antimicrobial resistance, not virulence. Microbial genomics. 2021 Feb;7(2).

For peer review only - http://bmjopen.bmj.com/site/about/guidelines.xhtml

Figures

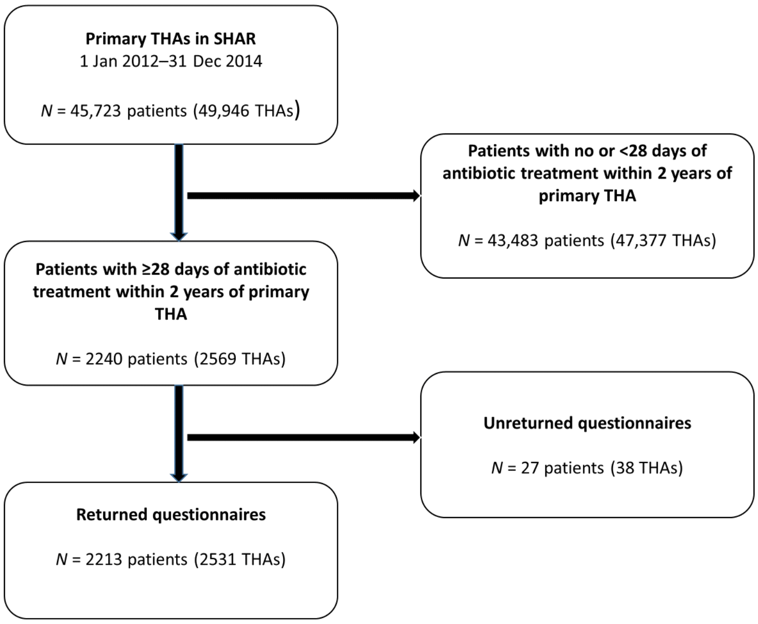

Figure 1. Study flowchart of primary THR with antibiotic treatment ≥28 days followed up for review of medical records.

review only

For peer review only - http://bmjopen.bmj.com/site/about/guidelines.xhtml

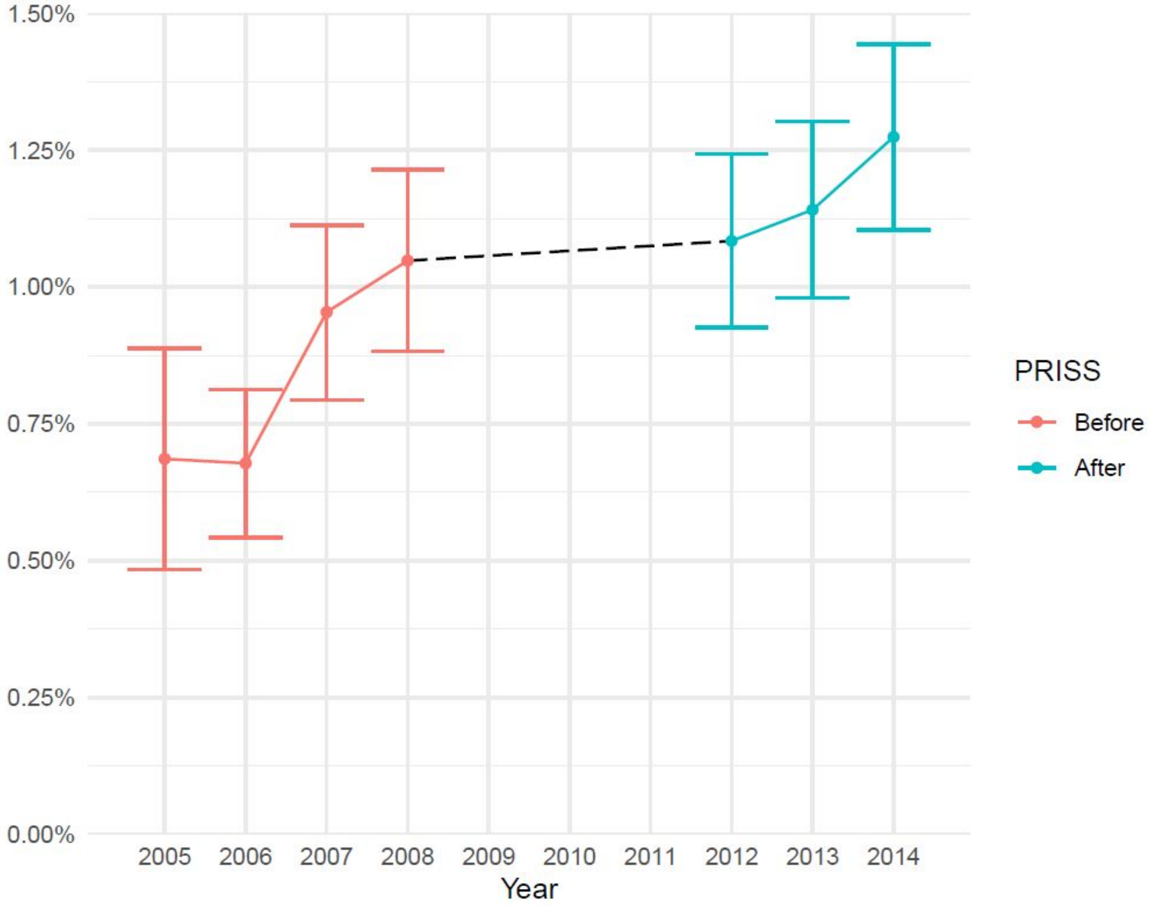

Figure 2. Trend of cumulative prosthetic joint infection incidence pre and post PRISS, 2005-2014.

For peer review only - http://bmjopen.bmj.com/site/about/guidelines.xhtml

For peer review only

For peer review only - http://bmjopen.bmj.com/site/about/guidelines.xhtml

Tables

Table 1. Examples of procedures and improvements addressed in the PRISS project, 2009–2012.

Pre-operative	Operation environment	Post-operative
No-smoking information, medicines	Improved hygiene routines, standardization of protocols	Wound treatment, protocol for changing dressing, sterile environment
BMI limit	CFU measurement (<5 CFU/m^3)	Early detection
Diabetic control	Locked operation room	Patient information, sign of infection, contact information
Hemoglobin levels	Maximum number of people in OR	
Patient information		Removing sutures at department
Skin-sanitizing procedures	Normothermia, regular check of body temperature	Early detection of infection, phone screening 1–2 weeks post-op
Full body examination	Improved and disposable clothing for all personnel	Follow-up instructions for primary health care
Clear criteria for cancellation, active infection	WHO checklist for follow-up improvement	
Preoperative decolonization	Antibiotic prophylaxis, type, timing, dose and intervals	

BMI, body mass index; CFU, colony forming units; WHO, World Health Organization.

For peer review only

For peer review only - http://bmjopen.bmj.com/site/about/guidelines.xhtml

Table 2. Demographic data for patients undergoing primary total hip arthroplasty, pre (2005–2008) and post PRISS (2012–2014).

	**Pre PRISS** (*n* = 49,259)	**Post PRISS** (*n* = 49,946)
Mean age (SD)	69 (11)	69 (11)
Female, sex	50 (29,078/49,259)	50 (28,645/49,946)
BMI ≥ 30	22 (2612/11,969)	24 (11,227/47,445)
ASA classification ≥ 3	17 (2252/13,069)	20 (9595/48,868)
Indication for operation		
Primary OA	83 (40,884/49,207)	80 (39,632/49,804)
Secondary OA	0 (22/49,207)	2 (1183/49,804)
Acute trauma, hip fracture	7 (3348/49,207)	9 (4325/49,804)
Complication trauma	3 (1413/49,207)	3 (1715/49,804)
Sequelae of childhood hip disease	2(1001/49,207)	2 (936/49,804)
Femoral head necrosis	3 (1235/49,207)	2 (1162/49,804)
Inflammatory joint disease	2 (1016/49,207)	1 (562/49,804)
Tumor	1 (288/49,207)	1 (291/49,804)
Surgical approach		
Direct lateral	44 (21,317/48,889)	48 (23,836/49,942)
Posterior	55 (26,786/48,889)	51 (25,654/49,942)
Other[1]	2 (786/ 48,889)	1 (454/49,942)
Implant fixation, any cemented[2]	89 (43,376/48,761)	82 (40,709/49,945)

Data shown as % (*n*) unless otherwise indicated.
BMI, body mass index; ASA American Society of Anesthesiology class, OA, osteoarthritis; PJI, prosthetic joint infection; MIS, minimally invasive surgery; SD, standard deviation.
[1] Trochanteric osteotomy, minimally invasive surgery.
[2] Cemented, hybrid, and reversed hybrid.

For peer review only

For peer review only - http://bmjopen.bmj.com/site/about/guidelines.xhtml

Table 3. Distribution of microorganisms isolated from prosthetic joint infections of the hip, pre (2005-2008) and post the PRISS project (2012-2014)

Pathogens	Pre PRISS (n=442)	Post PRISS (=580)
Streptococcus spp.	7% (32)	8% (44)
Staphylococcus spp.		
Staphylococcus aureus	20% (89)	26% (150[1])
Coagulase-negative staphylococci	24% (108)	21% (122)
Enterococcus spp.	4% (16)	3% (20)
Enterobacterales	3% (15)	4% (25)
Anaerobes	1% (3)	3% (17)
Miscellaneous[2]	3% (13)	1% (6)
Polymicrobial[3]	27% (120)	25% (146)
Unknown[4]	10% (46)	9% (50)

[1] Including one methicillin-resistant *Staphylococcus aureus*.
[2] e.g. *Bacillus, Corynebacterium, Mycobacterium, Pseudomonas.*
[3] Polymicrobial findings (2–4 organisms). Pre PRISS 100% of polymicrobial PJIs included *Staphylococcus spp*. vs. 82% post PRISS project.
[4] Culture-negative PJIs and missing data.

For peer review only

For peer review only

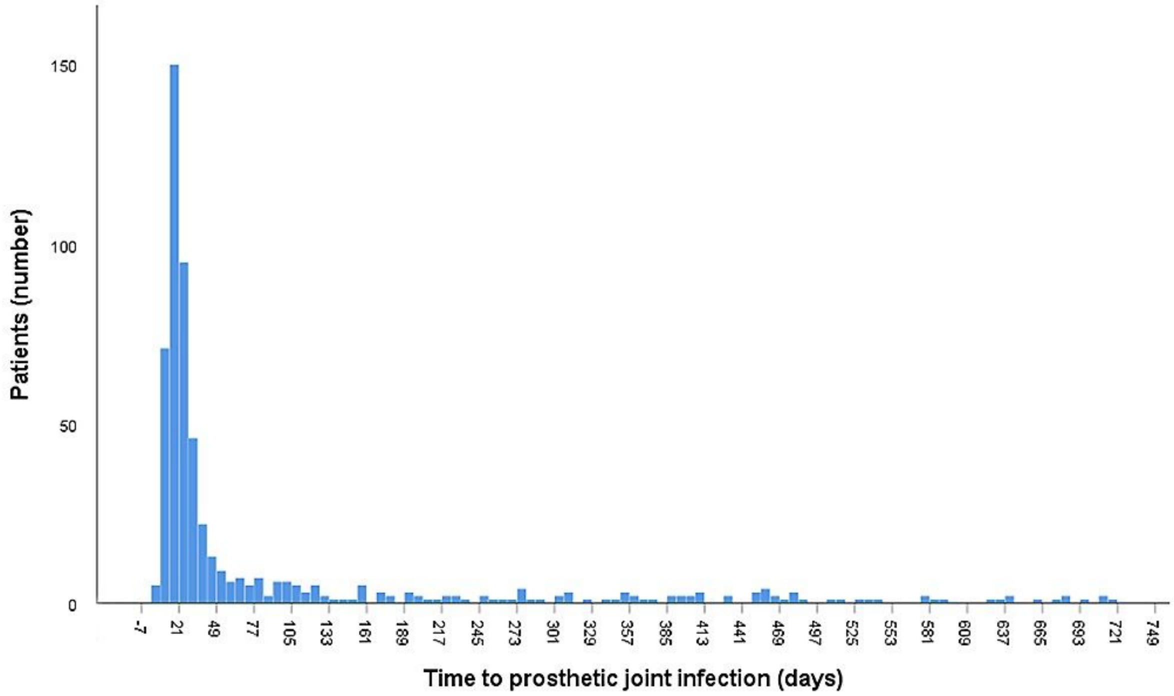

Supplemental Figure 1. Time from total hip arthroplasty to prosthetic joint infection diagnosis following the PRISS project, 2012–2014. Each bar represents 1 week.

Supplemental Table 1. Diagnostic criteria walkthrough for patients treated for PJI pre PRISS (2005-08) post PRISS (2012-14); diagnostic criteria were verified sequentially.

Diagnostic criteria	(n=442)	(n=580)
1. Sinus tract communicating with the prosthesis	14 (64)	29 (168)
2. ≥2 positive periprosthetic cultures with same pathogen	59 (262)	53 (310)
3a + 3b. Presence of purulence in joint + elevated CRP/SR	5 (23)	5 (30)
3a + 3c. Presence of purulence + isolation of one periprosthetic pathogen in tissue or fluid	4 (19)	5 (31)
3b + 3c. Elevated CRP/SR + isolation of one periprosthetic pathogen in tissue or fluid	7 (29)	2 (10)
Clinical diagnosis or lack of information in questionnaires	10 (45)	5 (31)

Data presented as % (n)

CRP, C-reactive protein; SR, sedimentation rate.

Supplemental Table 2. Characteristics of patients with and without prosthetic joint infection (PJI) within 2 years of primary total hip placement surgery, 2012–2014.

	**PJI** (n = 580)	**Control** (n = 49,366)	***p*–value**
Mean age (SD)	71 (10)	69 (10)	<0.001
Female, sex	41 (240)	58 (28,405)	<0.001
BMI ≥ 30	33 (178)	24 (11,049)	<0.001
ASA classification ≥ 3	35 (200)	19 (9395)	<0.001
Indication for operation			<0.001
Primary OA	70 (401)	80 (39,231)	
Secondary OA	4 (22)	2 (1161)	
Acute trauma	12 (70)	9 (4255)	
Complication trauma	7 (40)	3 (1675)	
Sequelae of childhood hip disease	1 (7)	2 (929)	
Femoral head necrosis	3 (18)	2 (1144)	
Inflammatory joint disease	1 (13)	1 (547)	
Tumor	1 (6)	1 (285)	
Surgical approach			0.37
Direct lateral	51 (295)	48 (23,541)	
Posterior	48 (280)	51 (25,374)	
Trochanteric Osteotomy	0 (0)	0 (70)	
MIS[1]	1 (4)	1 (365)	
Implant fixation			0.22
Cemented	69 (402)	66 (32,530)	
Hybrid/reversed-hybrid	14 (80)	19 (9138)	
Uncemented	17 (98)	19 (9138)	

Data shown as % (n) unless otherwise indicated.
BMI, body mass index; ASA American Society of Anesthesiology class, OA, osteoarthritis; PJI, prosthetic joint infection; MIS, minimally invasive surgery; SD, standard deviation.
[1] MIS anterior, MIS posterior, Watson-Jones.

For peer review only

For peer review only

STROBE Statement

Checklist of items that should be included in reports of observational studies

Section/Topic	Item No	Recommendation	Reported on Page No
**Title and abstract**	1	(*a*) Indicate the study's design with a commonly used term in the title or the abstract	1
		(*b*) Provide in the abstract an informative and balanced summary of what was done and what was found	3-4
**Introduction**			
Background/rationale	2	Explain the scientific background and rationale for the investigation being reported	4-5
Objectives	3	State specific objectives, including any prespecified hypotheses	5
**Methods**			
Study design	4	Present key elements of study design early in the paper	5
Setting	5	Describe the setting, locations, and relevant dates, including periods of recruitment, exposure, follow-up, and data collection	5-6
Participants	6	(*a*) *Cohort study*—Give the eligibility criteria, and the sources and methods of selection of participants. Describe methods of follow-up<br>*Case-control study*—Give the eligibility criteria, and the sources and methods of case ascertainment and control selection. Give the rationale for the choice of cases and controls<br>*Cross-sectional study*—Give the eligibility criteria, and the sources and methods of selection of participants	5-6
		(*b*) *Cohort study*—For matched studies, give matching criteria and number of exposed and unexposed<br>*Case-control study*—For matched studies, give matching criteria and the number of controls per case	
Variables	7	Clearly define all outcomes, exposures, predictors, potential confounders, and effect modifiers. Give diagnostic criteria, if applicable	5-7
Data sources/measurement	8*	For each variable of interest, give sources of data and details of methods of assessment (measurement). Describe comparability of assessment methods if there is more than one group	6,7
Bias	9	Describe any efforts to address potential sources of bias	9-11
Study size	10	Explain how the study size was arrived at	6,7
Quantitative variables	11	Explain how quantitative variables were handled in the analyses. If applicable, describe which groupings were chosen and why	7,8
Statistical methods	12	(*a*) Describe all statistical methods, including those used to control for confounding	7,8
		(*b*) Describe any methods used to examine subgroups and interactions	7,8
		(*c*) Explain how missing data were addressed	7,8
		(*d*) *Cohort study*—If applicable, explain how loss to follow-up was addressed<br>*Case-control study*—If applicable, explain how matching of cases and controls was addressed<br>*Cross-sectional study*—If applicable, describe analytical methods taking account of sampling strategy	7,8
		(*e*) Describe any sensitivity analyses	7,8

For peer review only - http://bmjopen.bmj.com/site/about/guidelines.xhtml

Section/Topic	Item No	Recommendation	Reported on Page No
**Results**			
Participants	13*	(a) Report numbers of individuals at each stage of study—eg numbers potentially eligible, examined for eligibility, confirmed eligible, included in the study, completing follow-up, and analysed	6,7
		(b) Give reasons for non-participation at each stage	
		(c) Consider use of a flow diagram	Figure 1
Descriptive data	14*	(a) Give characteristics of study participants (eg demographic, clinical, social) and information on exposures and potential confounders	Table 2
		(b) Indicate number of participants with missing data for each variable of interest	Figure 1
		(c) *Cohort study*—Summarise follow-up time (eg, average and total amount)	7
Outcome data	15*	*Cohort study*—Report numbers of outcome events or summary measures over time	8, Figure 2
		Case-control study—Report numbers in each exposure category, or summary measures of exposure	
		Cross-sectional study—Report numbers of outcome events or summary measures	
Main results	16	(*a*) Give unadjusted estimates and, if applicable, confounder-adjusted estimates and their precision (eg, 95% confidence interval). Make clear which confounders were adjusted for and why they were included	8
		(*b*) Report category boundaries when continuous variables were categorized	8
		(*c*) If relevant, consider translating estimates of relative risk into absolute risk for a meaningful time period	
Other analyses	17	Report other analyses done—eg analyses of subgroups and interactions, and sensitivity analyses	8,9
**Discussion**			
Key results	18		9
Limitations	19	Discuss limitations of the study, taking into account sources of potential bias or imprecision. Discuss both direction and magnitude of any potential bias	9-13
Interpretation	20	Give a cautious overall interpretation of results considering objectives, limitations, multiplicity of analyses, results from similar studies, and other relevant evidence	9-13
Generalisability	21	Discuss the generalisability (external validity) of the study results	13
**Other Information**			
Funding	22	Give the source of funding and the role of the funders for the present study and, if applicable, for the original study on which the present article is based	14

**Give information separately for cases and controls in case-control studies and, if applicable, for exposed and unexposed groups in cohort and cross-sectional studies.*

Note: An Explanation and Elaboration article discusses each checklist item and gives methodological background and published examples of transparent reporting. The STROBE checklist is best used in conjunction with this article (freely available on the Web sites of PLoS Medicine at http://www.plosmedicine.org/, Annals of Internal Medicine at http://www.annals.org/, and Epidemiology at http://www.epidem.com/). Information on the STROBE Initiative is available at www.strobe-statement.org.

For peer review only - http://bmjopen.bmj.com/site/about/guidelines.xhtml

BMJ Open

The effect of a national infection control program in Sweden on prosthetic joint infection incidence following primary total hip arthroplasty: a cohort study.

Journal:	*BMJ Open*
Manuscript ID	bmjopen-2023-076576.R1
Article Type:	Original research
Date Submitted by the Author:	26-Jan-2024
Complete List of Authors:	Wildeman, Peter; Orebro universitet, Department of Orthopedics, Faculty of Medicine and Health<br>Rolfson, Ola; University of Gothenburg, Department of Orthopaedics, Institute of Clinical Sciences, Sahlgrenska Academy; Registercentrum Vastra Gotaland, Swedish Arthroplasty Register<br>Wretenberg, Per; Örebro University, Department of Orthopedics, Faculty of Medicine and Health<br>Nåtman, Jonatan; Centre of Registers Vastra Gotaland, Swedish Arthroplasty Register<br>Gordon, Max; Karolinska Institute, Department of Clinical Sciences, Danderyd Hospital<br>Söderqvist, Bo; Örebro University, Department of Infectious Diseases, Faculty of Medicine and Health; Örebro University, School of Medical Sciences, Faculty of Medicine and Health<br>Lindgren, Viktor; Karolinska Institutet, Department of Molecular Medicine and Surgery
**Primary Subject Heading**:	Infectious diseases
Secondary Subject Heading:	Surgery
Keywords:	Hip < ORTHOPAEDIC & TRAUMA SURGERY, Infection control < INFECTIOUS DISEASES, Primary Prevention, Epidemiology < INFECTIOUS DISEASES

SCHOLARONE™
Manuscripts

I, the Submitting Author has the right to grant and does grant on behalf of all authors of the Work (as defined in the below author licence), an exclusive licence and/or a non-exclusive licence for contributions from authors who are: i) UK Crown employees; ii) where BMJ has agreed a CC-BY licence shall apply, and/or iii) in accordance with the terms applicable for US Federal Government officers or employees acting as part of their official duties; on a worldwide, perpetual, irrevocable, royalty-free basis to BMJ Publishing Group Ltd ("BMJ") its licensees and where the relevant Journal is co-owned by BMJ to the co-owners of the Journal, to publish the Work in this journal and any other BMJ products and to exploit all rights, as set out in our licence.

The Submitting Author accepts and understands that any supply made under these terms is made by BMJ to the Submitting Author unless you are acting as an employee on behalf of your employer or a postgraduate student of an affiliated institution which is paying any applicable article publishing charge ("APC") for Open Access articles. Where the Submitting Author wishes to make the Work available on an Open Access basis (and intends to pay the relevant APC), the terms of reuse of such Open Access shall be governed by a Creative Commons licence – details of these licences and which Creative Commons licence will apply to this Work are set out in our licence referred to above.

Other than as permitted in any relevant BMJ Author's Self Archiving Policies, I confirm this Work has not been accepted for publication elsewhere, is not being considered for publication elsewhere and does not duplicate material already published. I confirm all authors consent to publication of this Work and authorise the granting of this licence.

For peer review only

The effect of a national infection control program in Sweden on prosthetic joint infection incidence following primary total hip arthroplasty: a cohort study

Peter Wildeman [1,2], Ola Rolfson [3,4], Per Wretenberg [1,2], Jonatan Nåtman[4], Max Gordon [5], Bo Söderquist [2,6], and Viktor Lindgren[7]

1 Department of Orthopedics, Faculty of Medicine and Health, Örebro University, Örebro

2 School of Medical Sciences, Faculty of Medicine and Health, Örebro University, Örebro

3 Department of Orthopedics, Institute of Clinical Sciences, Sahlgrenska Academy, University of Gothenburg, Gothenburg

4 Swedish Arthroplasty Register, Centre of Registers, Västra Götalandsregionen, Gothenburg

5 Department of Clinical Sciences, Karolinska Institutet, Danderyd Hospital, Stockholm

6 Department of Infectious Diseases, Faculty of Medicine and Health, Örebro University, Örebro

7 Department of Molecular Medicine and Surgery, Karolinska Institutet, Stockholm, Sweden

Word count: Abstract 245

Manuscript, excluding abstract, 2859

Corresponding author:

Peter Wildeman

Department of Orthopedics

Örebro University Hospital

SE-70185 Örebro

For peer review only - http://bmjopen.bmj.com/site/about/guidelines.xhtml

Sweden

Telephone: +46196021000

peter.wildeman@regionorebrolan.se

For peer review only - http://bmjopen.bmj.com/site/about/guidelines.xhtml

Abstract

Objectives

Prosthetic joint infection (PJI) is a serious complication following total hip arthroplasty (THA) entailing increased mortality, decreased quality of life, and high healthcare costs.

The primary aim was to investigate whether the national project: Prosthesis Joint Infections shall be stopped (PRISS) reduced PJI incidence after primary THA; the secondary aim was to evaluate other possible benefits of PRISS, such as shorter time to diagnosis.

Design

Cohort study.

Setting

In 2009 a nationwide, multidisciplinary infection control program was launched in Sweden, PRISS, which aimed to reduce the PJI burden by 50%.

Participants

We obtained data on patients undergoing primary THA from the Swedish Arthroplasty Registry 2012–2014, (n = 45,723 patients, 49,946 THAs). Using personal identity numbers, this cohort was matched with the Swedish Prescribed Drug Registry. Medical records of patients with ≥4 weeks antibiotic consumption were reviewed to verify PJI diagnosis (n = 2240, 2569 THAs).

Results

The cumulative incidence of PJI following the PRISS project was 1.2% [95% CI 1.1-1.3] as compared to 0.9% [95% CI 0.8-1.0] before. Cox regression models for the PJI incidence post PRISS indicates there were no statistical significance difference versus pre PRISS (HR 1.1 [95% CI 0.9-1.3]. There were similar time to PJI diagnosis after the PRISS project 24 vs 23 days (p=0.5).

For peer review only

Conclusions

Despite the comprehensive nationwide PRISS project, Swedish PJI incidence was higher after the project and time to diagnosis remained unchanged. Factors contributing to PJI, such as increasing obesity, higher ASA class, and more fractures as indications, explain the PJI increase among primary THA patients.

Strengths and limitations of this study

- Utilized the exact same method for data capturing as a previous study investigating the PJI incidence in Sweden. Including several large national databases.
- Long follow-up time to capture PJI events and a very high response rate to medical record questionnaires, from all units operating total hip arthroplasty, in Sweden.
- We recognize the limitations of the study, as non-randomized, to establish causality.
- Not all risk factors possible influencing the outcome were included in the multivariate analysis and a possible underestimation of the PJI incidence due lack of ability to identify patients who died in hospital or long term intra hospital antibiotic treatment

Keywords: PJI, Prosthetic Joint Infection, Prevention, Arthroplasty, Hip.

For peer review only

For peer review only - http://bmjopen.bmj.com/site/about/guidelines.xhtml

Introduction

In a world with an increasing elderly population (1) whose members wish to live healthy and mobile lives for most of their lifespans, artificial hip joints improve quality of life (QoL) for millions of patients every year (2). Excellent long-term pain reduction and improved mobility have popularized the surgical procedure of total hip arthroplasty (THA). However, as with any surgical intervention, THA is associated with procedure-related complications. Aseptic loosening, repeated hip dislocation, thromboembolic event, and prosthetic joint infection (PJI) are the most common complications (3-5). PJIs have multifactorial causes, and extensive research has explored the risk factors for infection (3). Those associated with PJIs are both endogenous factors such as male gender, obesity, poor diabetic control, and active smoking (6, 7), and exogenous factors such as timing of prophylactic antibiotics, surgical site preparation, and laminar airflow (8-10). PJI as reason for reoperation has gradually increased in recent decades and is now the most common indication for reoperation within two years of primary THA (4). PJIs are associated with increased mortality (11-13), lower QoL (13, 14), and higher costs for healthcare providers (15, 16). In an attempt to reduce the PJI burden, the Swedish Orthopedic Association and Swedish National Patient Insurance started 2009 a nationwide, multidisciplinary infection control program: PRISS (Prosthetic Related Infections Shall be Stopped). All orthopedic surgical units (n = 81) in Sweden participated in the PRISS project and were reviewed. The goal of the project was to reduce PJI incidence following total hip and knee arthroplasty in Sweden by 50%. A study by Lindgren et al (17) estimated the PJI incidence to 0.9% after primary THA, the years prior to PRISS, 2005-2008. The effect of the PRISS project on PJI after THA is however unknown. The primary aim of the present study was therefore to investigate if the PRISS project reduced the incidence of PJI following THA; the secondary aims were to evaluate other possible benefits of the initiative, i.e., shorter time to diagnosis, and to identify pathogens involved in PJIs.

For peer review only - http://bmjopen.bmj.com/site/about/guidelines.xhtml

Methods and analysis

Design

The PRISS study is a cohort study.

Registries

All public and private orthopedic clinics performing THA operations report to the Swedish Arthroplasty Register (SAR). Approximately 98% of all THAs in Sweden are reported to the registry (4). Details such as indication for surgery, type of implant, surgical approach, body mass index (BMI), and American Society of Anesthesiology class (ASA) are reported. The Swedish Prescribed Drug Registry (SPDR) is operated by the Swedish National Board of Health and Welfare, and all pharmacies in Sweden automatically report complete details regarding dispensed drugs, including type of drug, amount dispensed, instructions from prescribing doctor, and dates of prescription and dispensing.

For peer review only

Swedish National Patient Insurance and the PRISS project

The Swedish National Patient Insurance (SNPI) is a public insurance company jointly owned by its policy holders, the Swedish regions and county councils. Its primary goals are to insure its owners, handle claims, compensate patients affected by health-care-associated complications, and address patient safety. In 2008, 33% of all claims were from the orthopedic specialty (18). In 2009,the PRISS project was initiated jointly by SNPI and the Swedish Orthopedic Association (18). The project started in 2009 and by 2012 all orthopedic units (n = 81) had been enrolled in it. It was an interdisciplinary project for safer THA and TKA operations involving several professional organizations such as the Swedish associations for orthopedic, infectious disease specialists, surgical nursing, orthopedic nursing, physical therapy, and infection control. A self-assessment tool, site inspections, and expert group evaluations were performed on all units to evaluate the routines before, during and after

arthroplasty surgery (18). Eighty-five percent of all implemented measures were improvement in procedures and protocols. Factors such as optimizing patients before surgery, basic hygiene routines, optimizing antibiotic prophylaxis, improving operating theatres (e.g. CFU measurements, locked rooms and maximum number of persons in operating rooms), and postoperative wound care were among those factors addressed (Table 1).

Table 1. Examples of procedures and improvements addressed in the PRISS project, 2009–2012.

Pre-operative	Operation environment	Post-operative
No-smoking information, medicines	Improved hygiene routines, standardization of protocols	Wound treatment, protocol for changing dressing, sterile environment
BMI limit	CFU measurement (<5 CFU/m^3)	Early detection
Diabetic control	Locked operation room	Patient information, sign of infection, contact information
Hemoglobin levels	Maximum number of people in OR	
Patient information		Removing sutures at department
Skin-sanitizing procedures	Normothermia, regular check of body temperature	Early detection of infection, phone screening 1–2 weeks post-op
Full body examination	Improved and disposable clothing for all personnel	Follow-up instructions for primary health care
Clear criteria for cancellation, active infection	WHO checklist for follow-up improvement	
Preoperative decolonization	Antibiotic prophylaxis, type, timing, dose and intervals	

BMI, body mass index; CFU, colony forming units; WHO, World Health Organization.

For peer review only

For peer review only - http://bmjopen.bmj.com/site/about/guidelines.xhtml

However, the impact of optimization of these measures in the setting of the PRISS project, on the PJI risk, has not been explored.

Study design, participants, and setting

A new method of postoperative infection surveillance introduced by Lindgren et al. combined national medical registry data with medical record review (17), determining PJI cumulative incidence within 2 years of primary THA to be 0.9% (95% CI 0.9-1.0) between 2005 and 2008. From that study, we extracted patient demographic data, on patients with a primary THA (all indications) and a PJI diagnosis to compare with the post PRISS cohort (Table 2).

Table 2. Demographic data for patients undergoing primary total hip arthroplasty, pre (2005–2008) and post PRISS (2012–2014).

	**Pre PRISS** (*n* = 49,259)	**Post PRISS** (*n* = 49,946)
Mean age (SD)	69 (11)	69 (11)
Female, sex	50 (29,078/49,259)	50 (28,645/49,946)
BMI ≥ 30	22 (2612/11,969)	24 (11,227/47,445)
ASA classification ≥ 3	17 (2252/13,069)	20 (9595/48,868)
Indication for operation		
Primary OA	83 (40,884/49,207)	80 (39,632/49,804)
Secondary OA	0 (22/49,207)	2 (1183/49,804)
Acute trauma, hip fracture	7 (3348/49,207)	9 (4325/49,804)
Complication trauma	3 (1413/49,207)	3 (1715/49,804)
Sequelae of childhood hip disease	2(1001/49,207)	2 (936/49,804)
Femoral head necrosis	3 (1235/49,207)	2 (1162/49,804)
Inflammatory joint disease	2 (1016/49,207)	1 (562/49,804)
Tumor	1 (288/49,207)	1 (291/49,804)
Surgical approach		
Direct lateral	44 (21,317/48,889)	48 (23,836/49,942)
Posterior	55 (26,786/48,889)	51 (25,654/49,942)
Other[1]	2 (786/ 48,889)	1 (454/49,942)
Implant fixation, any cemented[2]	89 (43,376/48,761)	82 (40,709/49,945)

Data shown as % (*n*) unless otherwise indicated.
BMI, body mass index; ASA American Society of Anesthesiology class, OA, osteoarthritis; PJI, prosthetic joint infection; MIS, minimally invasive surgery; SD, standard deviation.
[1] Trochanteric osteotomy, minimally invasive surgery.
[2] Cemented, hybrid, and reversed hybrid.

For peer review only - http://bmjopen.bmj.com/site/about/guidelines.xhtml

The present study used the same method, including diagnostic criteria for PJI (19), to determine the incidence of PJI within 2 years of primary THA over the 2012–2014 period. From SAR we extracted all primary THA procedures (*n* = 49,946 in 45,723 patients) between 1 January 2012 and 31 December 2014 (Figure 1). Using personal identity numbers, we matched this cohort with the SPDR and identified 2240 patients undergoing a total of 2569 primary THAs with continuous outpatient consumption of antibiotics for a minimum of 4 weeks minimum of 2 years after each primary THA. For each THA, a questionnaire was sent to the operating unit, where a physician reviewed the medical records of that patient to verify that he/she had been treated for PJI. From the questionnaire, information on date of diagnosis, clinical presentation, and surgical treatment was obtained (Supplemental Table 1).

Ninety-nine percent (*n* = 2531/2569) of the questionnaires were returned along with the microbiology results. PJI after revision THA as well as superficial infections were excluded.

Statistics

Continuous variables were presented as means (with standard deviations) and medians (with inter-quantile ranges), as appropriate. The χ^2 test and Fisher's exact test were used to compare categorical data such as sex, BMI ≥ 30, and ASA ≥ 3. The *t*-test was used to evaluate between-group differences in age. Cumulative incidence of PJI with 95 % confidence intervals were estimated using the Kaplan-Meier estimator. Hazard ratios with 95 % confidence intervals were estimated using Cox regression adjusted for age, sex, and diagnosis (OA/other). Patients were followed for two years from primary operation until the first PJI and censored for death and revision for other causes than PJI. A sensitivity analysis, in the part of the cohort with complete data, a Cox regression also adjusted for ASA and BMI were estimated.

For peer review only - http://bmjopen.bmj.com/site/about/guidelines.xhtml

All tests were two-tailed, and statistical significance was defined as a *p*-value < 0.05 or a 95% confidence interval (CI)) excluding an odds ratio (OR) equal to 1.00. Statistical analyses were performed using SPSS version 25 (IBM Corp., Armonk, NY, USA).

Results

Five hundred and eighty patients treated for a PJI were identified (Supplemental Table 2), resulting in a nationwide 2-year cumulative incidence of 1.2% [95% CI 1.1-1.3] over the 2012–2014 period as compared to 0.9% [95% CI 0.8-1.0] in the 2005-2008 period. There was an increasing trend of PJI incidence during the study period (Figure 2) and a significant increase of 30% during for the whole study period p<0.001. However, the multivariate Cox regression model indicated no statistically significant difference in the cumulative incidence pre- versus post-PRISS PJI (HR 1.1 [95% CI 0.9–1.3],).

The highest incidence was noted in university hospitals (1.5%), followed by regional (1.4%) and county and private hospitals (both 0.9%). After controlling for the potential confounders of age, sex, indication for surgery, BMI ≥ 30, and ASA ≥ 3, we found no statistically significant differences in PJI incidence among the different hospital classes. For individual orthopedic units, the cumulative incidence ranged from 0.0 to 5.9%; in two units the incidence had decreased and in three it had increased compared with before the PRISS project. The median time from primary THA to PJI diagnosis was 23 days (IQR 48) (Supplemental figure 1), not significantly different from the 24 days before the PRISS project (IQR 46, *p*=0.51).

In Table 3 the distribution of the microbial agents isolated from PJIs pre and post PRISS is presented.

Table 3. Distribution of microorganisms isolated from prosthetic joint infections of the hip, pre (2005-2008) and post the PRISS project (2012-2014)

For peer review only - http://bmjopen.bmj.com/site/about/guidelines.xhtml

**Pathogens**	**Pre PRISS (n=442)**	**Post PRISS (=580)**
Streptococcus spp.	7% (32)	8% (44)
Staphylococcus spp.		
Staphylococcus aureus	20% (89)	26% (150[1])
Coagulase-negative staphylococci	24% (108)	21% (122)
Enterococcus spp.	4% (16)	3% (20)
Enterobacterales	3% (15)	4% (25)
Anaerobes	1% (3)	3% (17)
Miscellaneous[2]	3% (13)	1% (6)
Polymicrobial[3]	27% (120)	25% (146)
Unknown[4]	10% (46)	9% (50)

[1] Including one methicillin-resistant *Staphylococcus aureus*.
[2] e.g. *Bacillus, Corynebacterium, Mycobacterium, Pseudomonas.*
[3] Polymicrobial findings (2–4 organisms). Pre PRISS 100% of polymicrobial PJIs included *Staphylococcus spp*. vs. 82% post PRISS project.
[4] Culture-negative PJIs and missing data.

For peer review only

For peer review only - http://bmjopen.bmj.com/site/about/guidelines.xhtml

The most common microorganisms, isolated in pure culture or as part of polymicrobial growth, were *Staphylococcus* spp. (72% pre vs 68% post, p=0.9). In pure culture, *Staphylococcus aureus*, was the most abundant species, with a minor increase post PRISS (20% vs 26%, p=0.04), and one isolate was found to be methicillin resistant (i.e., MRSA) vs. none pre PRISS. Coagulase-negative staphylococci (CoNS) were the second most commonly isolated group of bacteria (21% vs 24%, p=0.2). The number of polymicrobial PJIs, with 2–4 different bacterial species, did not differ significantly pre vs post PRISS (27% vs 25%, p=0.5).

Discussion

We investigated the effect of PRISS on PJI incidence in patients with primary THA in the years following the project (i.e., 2012–2014) with a follow-up period of 2 years. We also examined time to diagnosis and infecting microorganisms. We found a 30 % increase in PJI incidence after as compared to before the PRISS project, i.e. 1.2 vs. 0.9% (17), but there was no statistically significant difference, after controlling for confounders such as BMI, ASA, and indication for surgery. Time to diagnosis remained unchanged pre vs. post PRISS (i.e., median 23 vs. 24 days).

Different sources of error may have affected the PJI incidence level reported here. One source of error was that the specific reason for antibiotic prescription could not be established in all cases. For example, if a patient had moved from the county where s/he initially underwent surgery, local medical record review was impossible. Another limitation concerns identifying the patient cohort through SPDR. Since SPDR only handles outpatient prescriptions, patients who died in hospital or were hospitalized during the course of treatment could not be detected; however, the mortality rate is very low following arthroplasty (20), limiting this effect. According to the Swedish national guidelines (21), the advocated antibiotic treatment

For peer review only - http://bmjopen.bmj.com/site/about/guidelines.xhtml

for PJI is 2 weeks of intravenous and 10 weeks of oral antibiotic therapy. Inpatient care for such long time is rare in Sweden, which minimizes the immortal time bias. In addition, the sensitivity of the 2011 MSIS criteria has been questioned (22). All these limitations may serve to underestimate the true incidence of PJI. Nevertheless, the study was performed using the exact same methodology as in the prior study by Lindgren et al, (17) establishing the PJI incidence before PRISS, and having the same limitations in both studies makes the results comparable. The study was non-randomized thus limiting its power to establish causality. SAR started to collect BMI and ASA data in 2008, limiting the number of patients for whom this information is available. Over the study period we could identify a trend of increasing comorbidity of the patients (age, BMI, ASA class) and proportion of patients with hip fracture/secondary OA/sequelae after fracture.

As the cause of PJI is multifactorial any initiative to reduce the infection incidence must be broad and include all health care disciplines involved. The PRISS project included implementations of a bundle of actions to improve pre-, intra- and post-operative care following expert reviews(18). All recommendations were voluntary for the orthopaedic units, possibly limiting the effect of best-practice methods. It is also possible that there are unknown confounders contribution to an increase in PJI incidence that were not measured.

Most infections were diagnosed in the first two weeks following THA and infections were only sporadically identified after the first 90 days (Supplemental figure 3). This implies that most infections originate from the surgical procedure or during the early postoperative period, indicating that properly timing the first dose of prophylactic antibiotics as well as very careful wound care in the early postoperative period are essential. This also implies that the improved post-operative procedures and protocols of PRISS, such as wound treatment protocols, phone

For peer review only - http://bmjopen.bmj.com/site/about/guidelines.xhtml

screening, and patient information, had limited impact on PJI incidence. There was no change in time to diagnosis compared with before the PRISS project (i.e., 23 vs. 24 days). This time to diagnosis is likely acceptable, as infection treatment with debridement, antibiotics, and implant retention (DAIR) has a chance of successful outcome (i.e., infection eradication) if initiated within 28 days of infection for acute infections (21, 23-25). If a PJI has progressed for more than 4 weeks extensive surgery, i.e. one or two stage exchange is advocated for a successful outcome.

The risk of PJI following the first year of surgery is approximately 1% annually (3, 17), a risk that seemed to be unaffected by the PRISS project. Late infections include both those contracted during surgery or the early postoperative phase progressing to chronic PJIs, and acute hematogenous infections, which by definition could not be affected by the PRISS project measures. Although hematogenous infections can be treated successfully with a DAIR procedure within 3 weeks of infection onset (25), this treatment algorithm is less likely to be successful in a procedure for chronic PJI (24, 26, 27). In this study we have not tried to distinguish between the two types of late infections.

The PJI incidence before PRISS was lower at the beginning of the study period, and there was a trend of increasing incidence at the end of 2008(17). A trend of increasing PJI incidence could also be detected over the 2012–2014 period, representing a continuation of the increasing incidence noted later in the 2007–2008 period (Figure 2). This rising trend could have been because determination to achieve improvement diminished following the end of PRISS. The PRISS project was a national intervention with broad support from participants from all professional organizations in collaboration with SNPI and all orthopedic units. Despite broad support and an evidence-based approach, PRISS project did not reduce PJI

For peer review only - http://bmjopen.bmj.com/site/about/guidelines.xhtml

incidence. During the 2008–2014 period, the number of patients with severe systemic disease (ASA ≥ 3) increased, possibly contributing to the increasing PJI incidence over this period(28). More obese patients and patients with hip fractures underwent THA post PRISS (Table 2). Early operation is very important for a patient with a hip fracture (29) , but may also risk non-compliance to preoperative routines, thus contributing to the PJI risk. Thompson et al concluded that the PRISS project did not had any effect on PJI incidence after primary total knee arthroplasty (TKA) with similar pre and post PJI incidence(30), but did not explore the effect of co-morbidities . If the trend of increasing co-morbidities apply to the patients with TKA, this could imply that PRISS project could have had an effect on the PJI incidence for TKA (30) . However, fracture as indication for primary TKA is rare as compared to THA, limiting this effect on PJI incidence for TKA. Although PJI incidence has progressively increased over the years in Sweden, it is comparable to that reported in an international context (31, 32). Some risk factors for infection cannot be influenced (e.g., sex, age, and reason for THA), whereas some, although modifiable (e.g., heart failure, diabetes, and obesity), are hard to influence especially if the patient is in pain and has difficulty walking before THA, meaning that they were largely unaffected by the preoperative measures of the PRISS initiative. However, it is possible that the PJI incidence might have been even higher without the PRISS project and its various benefits, such as more patients receiving correct initial treatment and therefore being cured of infection (3, 30).

Staphylococci were by far the most common etiological agent of PJI, in agreement with international reports (33) and national observations before PRISS (17, 34). Since only one methicillin-resistant *S. aureus* (MRSA) case was found, the vast majority of *S. aureus* were susceptible to most antibiotics. However, on the contrary, many of the CoNS were not determined to species level, and *S. epidermidis*, the most common staphylococcal species

For peer review only - http://bmjopen.bmj.com/site/about/guidelines.xhtml

among CoNS causing PJI, are in contrast to *S. aureus* often multidrug resistant (35). Whether a pattern of increasing multidrug resistance could explain the increasing PJI incidence was not evaluated here.

Conclusions

The PRISS project did not reduce the PJI incidence and the time to diagnosis remained unchanged. This study indicates a lack of impact of non-mandatory recommended measures, despite being evidence based, to be implemented to reduce multifactorial complications such as PJI of the hip when the incidence is low. Higher proportions of obese, comorbid, and fracture patients likely explain the minor increase in PJI incidence, and it is possible that in modern health care, endogenous risk factors might affect the PJI incidence more than exogenous (and modifiable) risk factors. Further studies of patients with a high risk of PJI, such as fracture, revision surgery and comorbid patients, are warranted to reduce overall incidence of PJI and its severe complications. An increased focus on PJIs may have contributed to a better treatment outcome, but this needs further studies.

Ethical approval

This study was approved by the Regional Ethical Review Board of Gothenburg, Sweden (reference number 2017/329-17).

Conflicts of interests

The authors declare they have no conflicts of interests.

For peer review only

For peer review only - http://bmjopen.bmj.com/site/about/guidelines.xhtml

Author contributions

All authors designed the study. VL had project supervision. PW, MG and JN analyzed the data. PW wrote the manuscript with contributions from OR, PWR, MG, JN, BS, and VL. All authors approved the final version of the manuscript.

Acknowledgments

The authors would like to thank all orthopedic units and SAR contact physicians for review of the medical records. The authors would also like to thank Ann-Britt Wall, Department of Orthopedics Region Örebro County, for administrative support.

Funding

The authors would like to thank the funding organizations, Research Committee of Örebro University, Region Örebro County (Grant no: OLL-917121), Sweden and Patientförsäkringen LÖF, Sweden (Grant no:NA), for financial support of the study. The funders had no role in study design, data collection and analysis, decision to publish or preparation of the manuscript.

For peer review only

Data sharing

All data relevant to the study are included in the article or uploaded as supplementary information.

Figures captions

Figure 1. Study flowchart of primary THR with antibiotic treatment ≥28 days followed up for review of medical records.

Figure 2. Trend of cumulative prosthetic joint infection incidence pre and post PRISS, 2005-2014.

For peer review only - http://bmjopen.bmj.com/site/about/guidelines.xhtml

Supplemental Figure 1. Time from total hip arthroplasty to prosthetic joint infection diagnosis following the PRISS project, 2012–2014. Each bar represents 1 week.

For peer review only

For peer review only - http://bmjopen.bmj.com/site/about/guidelines.xhtml

References

1. Ritchie H, Roser M. Age Structure. 2019 [cited 2021 04/20]; Available from: https://ourworldindata.org/age-structure.

2. Learmonth ID, Young C, Rorabeck C. The operation of the century: total hip replacement. Lancet. 2007 Oct 27;370(9597):1508-19.

3. Tande AJ, Patel R. Prosthetic joint infection. Clin Microbiol Rev. 2014 Apr;27(2):302-45.

4. Kärrholm J, Rogmark C, Nauclér E, Vinblad J, Mohaddes M, Rolfson O. Swedish Hip Arthroplasty Register Annual Report 2018. . 2018;2018(1st January).

5. Healy WL, Iorio R, Clair AJ, Pellegrini VD, Della Valle CJ, Berend KR. Complications of Total Hip Arthroplasty: Standardized List, Definitions, and Stratification Developed by The Hip Society. Clin Orthop Relat Res. 2016 Feb;474(2):357-64.

6. Iannotti F, Prati P, Fidanza A, Iorio R, Ferretti A, Pèrez Prieto D, et al. Prevention of Periprosthetic Joint Infection (PJI): A Clinical Practice Protocol in High-Risk Patients. Trop Med Infect Dis. 2020 Dec 11;5(4).

7. Sørensen LT. Wound healing and infection in surgery. The clinical impact of smoking and smoking cessation: a systematic review and meta-analysis. Arch Surg. 2012 Apr;147(4):373-83.

For peer review only

8. Alamanda VK, Springer BD. The prevention of infection: 12 modifiable risk factors. Bone Joint J. 2019 Jan;101-b(1_Supple_A):3-9.

9. Lenguerrand E, Whitehouse MR, Beswick AD, Kunutsor SK, Burston B, Porter M, et al. Risk factors associated with revision for prosthetic joint infection after hip replacement: a prospective observational cohort study. Lancet Infect Dis. 2018 Sep;18(9):1004-14.

10. Dale H, Fenstad AM, Hallan G, Havelin LI, Furnes O, Overgaard S, et al. Increasing risk of prosthetic joint infection after total hip arthroplasty. Acta Orthop. 2012 Oct;83(5):449-58.

11. Natsuhara KM, Shelton TJ, Meehan JP, Lum ZC. Mortality during total hip periprosthetic joint infection. J Arthroplasty. 2019 Jul;34(7s):S337-s42.

12. Gundtoft PH, Pedersen AB, Varnum C, Overgaard S. Increased Mortality After Prosthetic Joint Infection in Primary THA. Clin Orthop Relat Res. 2017 Nov;475(11):2623-31.

13. Wildeman P, Rolfson O, Söderquist B, Wretenberg P, Lindgren V. What Are the Long-term Outcomes of Mortality, Quality of Life, and Hip Function after Prosthetic Joint Infection of the Hip? A 10-year Follow-up from Sweden. Clin Orthop Relat Res. 2021 May 31.

14. Poulsen NR, Mechlenburg I, Soballe K, Lange J. Patient-reported quality of life and hip function after 2-stage revision of

For peer review only - http://bmjopen.bmj.com/site/about/guidelines.xhtml

chronic periprosthetic hip joint infection: a cross-sectional study. Hip Int. 2018 Jul;28(4):407-14.

15. Peel TN, Cheng AC, Lorenzo YP, Kong DC, Buising KL, Choong PF. Factors influencing the cost of prosthetic joint infection treatment. J Hosp Infect. 2013 Nov;85(3):213-9.

16. Lora-Tamayo J, Senneville É, Ribera A, Bernard L, Dupon M, Zeller V, et al. The Not-So-Good Prognosis of Streptococcal Periprosthetic Joint Infection Managed by Implant Retention: The Results of a Large Multicenter Study. Clin Infect Dis. 2017 Jun 15;64(12):1742-52.

17. Lindgren V, Gordon M, Wretenberg P, Karrholm J, Garellick G. Deep infection after total hip replacement: a method for national incidence surveillance. Infect Control Hosp Epidemiol. 2014 Dec;35(12):1491-6.

18. Gustafson P, Schultz T, Stefansdottir A. PRISS – ProsthesisRelated Infections Shall be Stopped 2014.

19. Parvizi J, Zmistowski B, Berbari EF, Bauer TW, Springer BD, Della Valle CJ, et al. New definition for periprosthetic joint infection: from the Workgroup of the Musculoskeletal Infection Society. Clin Orthop Relat Res. 2011 Nov;469(11):2992-4.

20. Dale H, Børsheim S, Kristensen TB, Fenstad AM, Gjertsen JE, Hallan G, et al. Perioperative, short-, and long-term mortality related to fixation in primary total hip arthroplasty: a study on 79,557 patients in the -Norwegian Arthroplasty Register. Acta Orthop. 2020 Apr;91(2):152-8.

For peer review only - http://bmjopen.bmj.com/site/about/guidelines.xhtml

21. Swedish Society for Infectious Diseases. Vårdprogram för Led- och skelettinfektioner 2018. https://infektion.net/2018; 128]. Available from: https://infektion.net/wp-content/uploads/2018/11/2018-vardprogram-led-och-skelettinfektioner-final-2018-11-29.pdf.

22. Koh IJ, Cho WS, Choi NY, Parvizi J, Kim TK. How accurate are orthopedic surgeons in diagnosing periprosthetic joint infection after total knee arthroplasty?: A multicenter study. Knee. 2015 Jun;22(3):180-5.

23. Osmon DR, Berbari EF, Berendt AR, Lew D, Zimmerli W, Steckelberg JM, et al. Executive summary: diagnosis and management of prosthetic joint infection: clinical practice guidelines by the Infectious Diseases Society of America. Clin Infect Dis. 2013 Jan;56(1):1-10.

24. Zimmerli W. Clinical presentation and treatment of orthopaedic implant-associated infection. J Intern Med. 2014 Aug;276(2):111-9.

25. Wouthuyzen-Bakker M, Sebillotte M, Lomas J, Taylor A, Palomares EB, Murillo O, et al. Clinical outcome and risk factors for failure in late acute prosthetic joint infections treated with debridement and implant retention. J Infect. 2019 Jan;78(1):40-7.

26. Wouthuyzen-Bakker M, Sebillotte M, Lomas J, Kendrick B, Palomares EB, Murillo O, et al. Timing of implant-removal in

For peer review only - http://bmjopen.bmj.com/site/about/guidelines.xhtml

late acute periprosthetic joint infection: A multicenter observational study. J Infect. 2019 Sep;79(3):199-205.

27. Maillet M, Pavese P, Bruley D, Seigneurin A, François P. Is prosthesis retention effective for chronic infections in hip arthroplasties? A systematic literature review. Eur J Clin Microbiol Infect Dis. 2015 Aug;34(8):1495-502.

28. Kärrholm J, Lindahl H, Malchau H, Mohaddes M, Rogmark C, Rolfson O. The Swedish Hip Arthroplasty Register Annual Report 2015. 2016.

29. Simunovic N, Devereaux Pj Fau - Sprague S, Sprague S Fau - Guyatt GH, Guyatt Gh Fau - Schemitsch E, Schemitsch E Fau - Debeer J, Debeer J Fau - Bhandari M, et al. Effect of early surgery after hip fracture on mortality and complications: systematic review and meta-analysis. (1488-2329 (Electronic)).

30. Thompson O, A WD, Lindgren V, Gordon M, Robertsson O, Stefánsdóttir A. Similar periprosthetic joint infection rates after and before a national infection control program: a study of 45,438 primary total knee arthroplasties. Acta Orthop. 2022 Jan 3;93:3-10.

31. Gundtoft PH, Pedersen AB, Schønheyder HC, Møller JK, Overgaard S. One-year incidence of prosthetic joint infection in total hip arthroplasty: a cohort study with linkage of the Danish Hip Arthroplasty Register and Danish Microbiology Databases. (1522-9653 (Electronic)).

For peer review only - http://bmjopen.bmj.com/site/about/guidelines.xhtml

32. McMaster Arthroplasty Collaborative (MAC). Risk Factors for Periprosthetic Joint Infection Following Primary Total Hip Arthroplasty: A 15-Year, Population-Based Cohort Study. J Bone Joint Surg Am. 2020;2020;102(6):503-509(1535-1386 (Electronic)).

33. Flurin L, Greenwood-Quaintance KE, Patel R. Microbiology of polymicrobial prosthetic joint infection. Diagn Microbiol Infect Dis. 2019 Jul;94(3):255-9.

34. Stefánsdóttir A, Johansson D, Knutson K, Lidgren L, Robertsson O. Microbiology of the infected knee arthroplasty: report from the Swedish Knee Arthroplasty Register on 426 surgically revised cases. Scand J Infect Dis. 2009;41(11-12):831-40.

35. Månsson E, Bech Johannesen T, Nilsdotter-Augustinsson Å, Söderquist B, Stegger M. Comparative genomics of Staphylococcus epidermidis from prosthetic-joint infections and nares highlights genetic traits associated with antimicrobial resistance, not virulence. Microbial genomics. 2021 Feb;7(2).

For peer review only - http://bmjopen.bmj.com/site/about/guidelines.xhtml

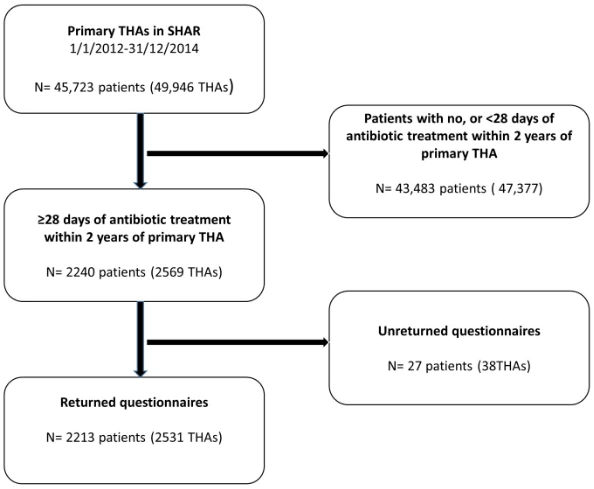

Figure 1. Study flowchart of primary THR with antibiotic treatment ≥28 days followed up for review of medical records.

338x190mm (300 x 300 DPI)

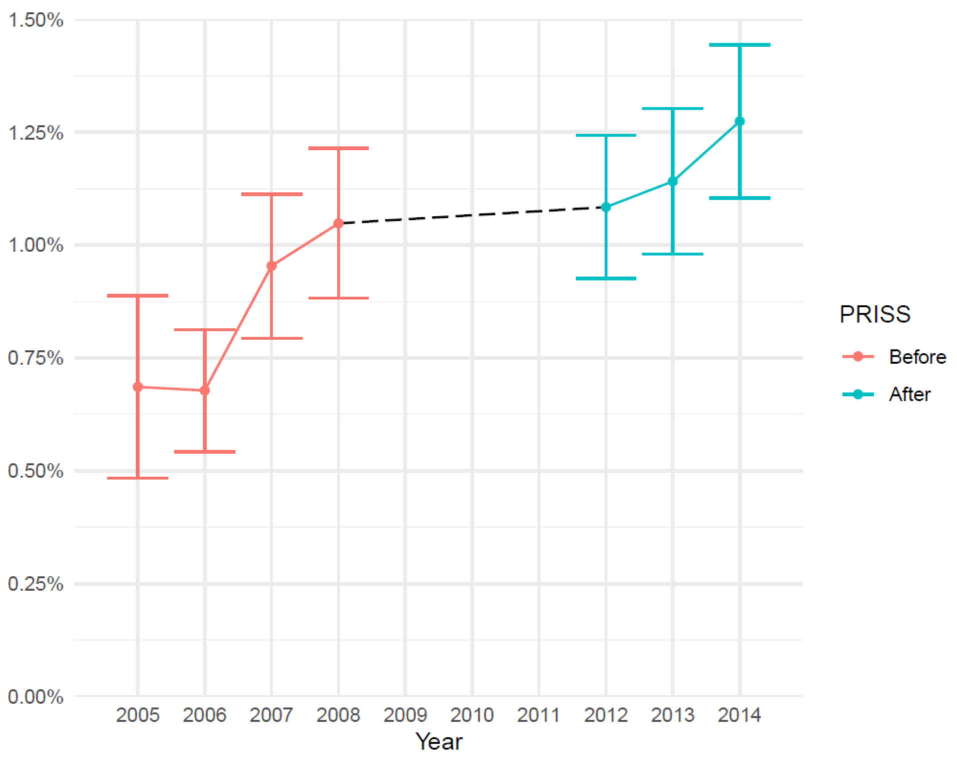

Figure 2. Trend of cumulative prosthetic joint infection incidence pre and post PRISS, 2005-2014.

88x66mm (300 x 300 DPI)

Supplemental Table 1. Diagnostic criteria walkthrough for patients treated for PJI pre PRISS (2005-08) post PRISS (2012-14); diagnostic criteria were verified sequentially.

Diagnostic criteria	(n=442)	(n=580)
1. Sinus tract communicating with the prosthesis	14 (64)	29 (168)
2. ≥2 positive periprosthetic cultures with same pathogen	59 (262)	53 (310)
3a + 3b. Presence of purulence in joint + elevated CRP/SR	5 (23)	5 (30)
3a + 3c. Presence of purulence + isolation of one periprosthetic pathogen in tissue or fluid	4 (19)	5 (31)
3b + 3c. Elevated CRP/SR + isolation of one periprosthetic pathogen in tissue or fluid	7 (29)	2 (10)
Clinical diagnosis or lack of information in questionnaires	10 (45)	5 (31)

Data presented as % (n)

CRP, C-reactive protein; SR, sedimentation rate.

Supplemental Table 2. Characteristics of patients with and without prosthetic joint infection (PJI) within 2 years of primary total hip placement surgery, 2012–2014.

	**PJI** (n = 580)	**Control** (n = 49,366)	***p*–value**
Mean age (SD)	71 (10)	69 (10)	<0.001
Female, sex	41 (240)	58 (28,405)	<0.001
BMI ≥ 30	33 (178)	24 (11,049)	<0.001
ASA classification ≥ 3	35 (200)	19 (9395)	<0.001
Indication for operation			<0.001
Primary OA	70 (401)	80 (39,231)	
Secondary OA	4 (22)	2 (1161)	
Acute trauma	12 (70)	9 (4255)	
Complication trauma	7 (40)	3 (1675)	
Sequelae of childhood hip disease	1 (7)	2 (929)	
Femoral head necrosis	3 (18)	2 (1144)	
Inflammatory joint disease	1 (13)	1 (547)	
Tumor	1 (6)	1 (285)	
Surgical approach			0.37
Direct lateral	51 (295)	48 (23,541)	
Posterior	48 (280)	51 (25,374)	
Trochanteric Osteotomy	0 (0)	0 (70)	
MIS[1]	1 (4)	1 (365)	
Implant fixation			0.22
Cemented	69 (402)	66 (32,530)	
Hybrid/reversed-hybrid	14 (80)	19 (9138)	
Uncemented	17 (98)	19 (9138)	

Data shown as % (n) unless otherwise indicated.

BMI, body mass index; ASA American Society of Anesthesiology class, OA, osteoarthritis; PJI, prosthetic joint infection; MIS, minimally invasive surgery; SD, standard deviation.

[1] MIS anterior, MIS posterior, Watson-Jones.

For peer review only

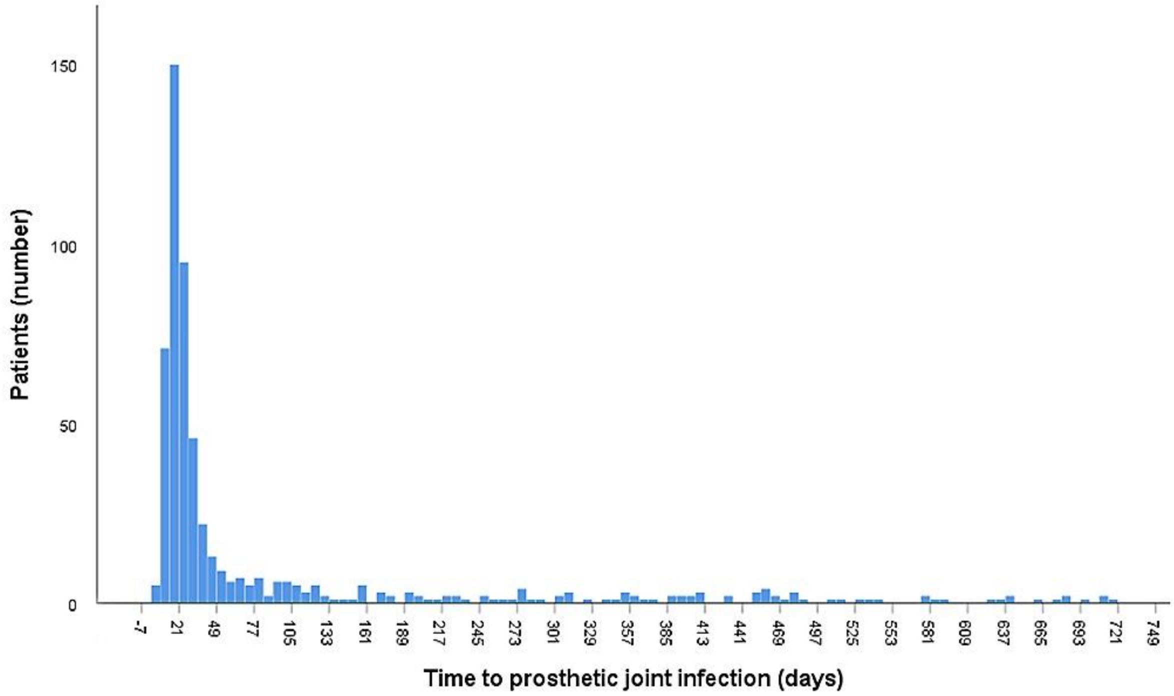

Supplemental Figure 1. Time from total hip arthroplasty to prosthetic joint infection diagnosis following the PRISS project, 2012–2014. Each bar represents 1 week.

For peer review only

STROBE Statement

Checklist of items that should be included in reports of observational studies

Section/Topic	Item No	Recommendation	Reported on Page No
**Title and abstract**	1	(*a*) Indicate the study's design with a commonly used term in the title or the abstract	1
		(*b*) Provide in the abstract an informative and balanced summary of what was done and what was found	3-4
**Introduction**			
Background/rationale	2	Explain the scientific background and rationale for the investigation being reported	4-5
Objectives	3	State specific objectives, including any prespecified hypotheses	5
**Methods**			
Study design	4	Present key elements of study design early in the paper	5
Setting	5	Describe the setting, locations, and relevant dates, including periods of recruitment, exposure, follow-up, and data collection	5-6
Participants	6	(*a*) *Cohort study*—Give the eligibility criteria, and the sources and methods of selection of participants. Describe methods of follow-up<br>*Case-control study*—Give the eligibility criteria, and the sources and methods of case ascertainment and control selection. Give the rationale for the choice of cases and controls<br>*Cross-sectional study*—Give the eligibility criteria, and the sources and methods of selection of participants	5-6
		(*b*) *Cohort study*—For matched studies, give matching criteria and number of exposed and unexposed<br>*Case-control study*—For matched studies, give matching criteria and the number of controls per case	
Variables	7	Clearly define all outcomes, exposures, predictors, potential confounders, and effect modifiers. Give diagnostic criteria, if applicable	5-7
Data sources/measurement	8*	For each variable of interest, give sources of data and details of methods of assessment (measurement). Describe comparability of assessment methods if there is more than one group	6,7
Bias	9	Describe any efforts to address potential sources of bias	9-11
Study size	10	Explain how the study size was arrived at	6,7
Quantitative variables	11	Explain how quantitative variables were handled in the analyses. If applicable, describe which groupings were chosen and why	7,8
Statistical methods	12	(*a*) Describe all statistical methods, including those used to control for confounding	7,8
		(*b*) Describe any methods used to examine subgroups and interactions	7,8
		(*c*) Explain how missing data were addressed	7,8
		(*d*) *Cohort study*—If applicable, explain how loss to follow-up was addressed<br>*Case-control study*—If applicable, explain how matching of cases and controls was addressed<br>*Cross-sectional study*—If applicable, describe analytical methods taking account of sampling strategy	7,8
		(*e*) Describe any sensitivity analyses	7,8

For peer review only - http://bmjopen.bmj.com/site/about/guidelines.xhtml

Section/Topic	Item No	Recommendation	Reported on Page No
**Results**			
Participants	13*	(a) Report numbers of individuals at each stage of study—eg numbers potentially eligible, examined for eligibility, confirmed eligible, included in the study, completing follow-up, and analysed	6,7
		(b) Give reasons for non-participation at each stage	
		(c) Consider use of a flow diagram	Figure 1
Descriptive data	14*	(a) Give characteristics of study participants (eg demographic, clinical, social) and information on exposures and potential confounders	Table 2
		(b) Indicate number of participants with missing data for each variable of interest	Figure 1
		(c) *Cohort study*—Summarise follow-up time (eg, average and total amount)	7
Outcome data	15*	*Cohort study*—Report numbers of outcome events or summary measures over time	8, Figure 2
		Case-control study—Report numbers in each exposure category, or summary measures of exposure	
		Cross-sectional study—Report numbers of outcome events or summary measures	
Main results	16	(*a*) Give unadjusted estimates and, if applicable, confounder-adjusted estimates and their precision (eg, 95% confidence interval). Make clear which confounders were adjusted for and why they were included	8
		(*b*) Report category boundaries when continuous variables were categorized	8
		(*c*) If relevant, consider translating estimates of relative risk into absolute risk for a meaningful time period	
Other analyses	17	Report other analyses done—eg analyses of subgroups and interactions, and sensitivity analyses	8,9
**Discussion**			
Key results	18		9
Limitations	19	Discuss limitations of the study, taking into account sources of potential bias or imprecision. Discuss both direction and magnitude of any potential bias	9-13
Interpretation	20	Give a cautious overall interpretation of results considering objectives, limitations, multiplicity of analyses, results from similar studies, and other relevant evidence	9-13
Generalisability	21	Discuss the generalisability (external validity) of the study results	13
**Other Information**			
Funding	22	Give the source of funding and the role of the funders for the present study and, if applicable, for the original study on which the present article is based	14

**Give information separately for cases and controls in case-control studies and, if applicable, for exposed and unexposed groups in cohort and cross-sectional studies.*

Note: An Explanation and Elaboration article discusses each checklist item and gives methodological background and published examples of transparent reporting. The STROBE checklist is best used in conjunction with this article (freely available on the Web sites of PLoS Medicine at http://www.plosmedicine.org/, Annals of Internal Medicine at http://www.annals.org/, and Epidemiology at http://www.epidem.com/). Information on the STROBE Initiative is available at www.strobe-statement.org.

For peer review only - http://bmjopen.bmj.com/site/about/guidelines.xhtml

BMJ Open

The effect of a national infection control program in Sweden on prosthetic joint infection incidence following primary total hip arthroplasty: a cohort study.

Journal:	*BMJ Open*
Manuscript ID	bmjopen-2023-076576.R2
Article Type:	Original research
Date Submitted by the Author:	29-Feb-2024
Complete List of Authors:	Wildeman, Peter; Orebro universitet, Department of Orthopedics, Faculty of Medicine and Health<br>Rolfson, Ola; University of Gothenburg, Department of Orthopaedics, Institute of Clinical Sciences, Sahlgrenska Academy; Registercentrum Vastra Gotaland, Swedish Arthroplasty Register<br>Wretenberg, Per; Örebro University, Department of Orthopedics, Faculty of Medicine and Health<br>Nåtman, Jonatan; Centre of Registers Vastra Gotaland, Swedish Arthroplasty Register<br>Gordon, Max; Karolinska Institute, Department of Clinical Sciences, Danderyd Hospital<br>Söderquist, Bo; Örebro University, School of Medical Sciences, Faculty of Medicine and Health; Örebro universitet, Department of Infectious Diseases, Faculty of Medicine and Health<br>Lindgren, Viktor; Karolinska Institutet, Department of Molecular Medicine and Surgery
**Primary Subject Heading**:	Infectious diseases
Secondary Subject Heading:	Surgery
Keywords:	Hip < ORTHOPAEDIC & TRAUMA SURGERY, Infection control < INFECTIOUS DISEASES, Primary Prevention, Epidemiology < INFECTIOUS DISEASES

SCHOLARONE™
Manuscripts

I, the Submitting Author has the right to grant and does grant on behalf of all authors of the Work (as defined in the below author licence), an exclusive licence and/or a non-exclusive licence for contributions from authors who are: i) UK Crown employees; ii) where BMJ has agreed a CC-BY licence shall apply, and/or iii) in accordance with the terms applicable for US Federal Government officers or employees acting as part of their official duties; on a worldwide, perpetual, irrevocable, royalty-free basis to BMJ Publishing Group Ltd ("BMJ") its licensees and where the relevant Journal is co-owned by BMJ to the co-owners of the Journal, to publish the Work in this journal and any other BMJ products and to exploit all rights, as set out in our licence.

The Submitting Author accepts and understands that any supply made under these terms is made by BMJ to the Submitting Author unless you are acting as an employee on behalf of your employer or a postgraduate student of an affiliated institution which is paying any applicable article publishing charge ("APC") for Open Access articles. Where the Submitting Author wishes to make the Work available on an Open Access basis (and intends to pay the relevant APC), the terms of reuse of such Open Access shall be governed by a Creative Commons licence – details of these licences and which Creative Commons licence will apply to this Work are set out in our licence referred to above.

Other than as permitted in any relevant BMJ Author's Self Archiving Policies, I confirm this Work has not been accepted for publication elsewhere, is not being considered for publication elsewhere and does not duplicate material already published. I confirm all authors consent to publication of this Work and authorise the granting of this licence.

For peer review only

The effect of a national infection control program in Sweden on prosthetic joint infection incidence following primary total hip arthroplasty: a cohort study

Peter Wildeman [1,2], Ola Rolfson [3,4], Per Wretenberg [1,2], Jonatan Nåtman[4], Max Gordon [5], Bo Söderquist [2,6], and Viktor Lindgren[7]

1 Department of Orthopedics, Faculty of Medicine and Health, Örebro University, Örebro

2 School of Medical Sciences, Faculty of Medicine and Health, Örebro University, Örebro

3 Department of Orthopedics, Institute of Clinical Sciences, Sahlgrenska Academy, University of Gothenburg, Gothenburg

4 Swedish Arthroplasty Register, Centre of Registers, Västra Götalandsregionen, Gothenburg

5 Department of Clinical Sciences, Karolinska Institutet, Danderyd Hospital, Stockholm

6 Department of Infectious Diseases, Faculty of Medicine and Health, Örebro University, Örebro

7 Department of Molecular Medicine and Surgery, Karolinska Institutet, Stockholm, Sweden

Word count: Abstract 245

Manuscript, excluding abstract, 2859

Corresponding author:

Peter Wildeman

Department of Orthopedics

Örebro University Hospital

SE-70185 Örebro

For peer review only - http://bmjopen.bmj.com/site/about/guidelines.xhtml

24 Sweden

25 Telephone: +46196021000

26 peter.wildeman@regionorebrolan.se

For peer review only

Abstract

Objectives

Prosthetic joint infection (PJI) is a serious complication following total hip arthroplasty (THA) entailing increased mortality, decreased quality of life, and high healthcare costs.

The primary aim was to investigate whether the national project: Prosthesis Joint Infections shall be stopped (PRISS) reduced PJI incidence after primary THA; the secondary aim was to evaluate other possible benefits of PRISS, such as shorter time to diagnosis.

Design

Cohort study.

Setting

In 2009 a nationwide, multidisciplinary infection control program was launched in Sweden, PRISS, which aimed to reduce the PJI burden by 50%.

Participants

We obtained data on patients undergoing primary THA from the Swedish Arthroplasty Registry 2012–2014, (n = 45,723 patients, 49,946 THAs). Using personal identity numbers, this cohort was matched with the Swedish Prescribed Drug Registry. Medical records of patients with ≥4 weeks antibiotic consumption were reviewed to verify PJI diagnosis (n = 2240, 2569 THAs).

Results

The cumulative incidence of PJI following the PRISS project was 1.2% [95% CI 1.1-1.3] as compared to 0.9% [95% CI 0.8-1.0] before. Cox regression models for the PJI incidence post PRISS indicates there were no statistical significance difference versus pre PRISS (HR 1.1 [95% CI 0.9-1.3]. There were similar time to PJI diagnosis after the PRISS project 24 vs 23 days (*p*=0.5).

For peer review only - http://bmjopen.bmj.com/site/about/guidelines.xhtml

Conclusions

Despite the comprehensive nationwide PRISS project, Swedish PJI incidence was higher after the project and time to diagnosis remained unchanged. Factors contributing to PJI, such as increasing obesity, higher ASA class, and more fractures as indications, explain the PJI increase among primary THA patients.

Strengths and limitations of this study

- Long follow-up time to capture PJI events and a very high response rate to questionnaires from all units in Sweden operating total hip arthroplasty.
- Utilized the exact same method for data capturing as a previous study investigating the PJI incidence in Sweden.
- We recognize the limitations of the study, as non-randomized, to establish causality.
- Not all risk factors possible influencing the outcome were included in the multivariate analysis and a possible underestimation of the PJI incidence due lack of ability to identify patients who died in hospital or long term intra hospital antibiotic treatment

Keywords: PJI, Prosthetic Joint Infection, Prevention, Arthroplasty, Hip.

For peer review only

For peer review only - http://bmjopen.bmj.com/site/about/guidelines.xhtml

Introduction

In a world with an increasing elderly population (1) whose members wish to live healthy and mobile lives for most of their lifespans, artificial hip joints improve quality of life (QoL) for millions of patients every year (2). Excellent long-term pain reduction and improved mobility have popularized the surgical procedure of total hip arthroplasty (THA). However, as with any surgical intervention, THA is associated with procedure-related complications. Aseptic loosening, repeated hip dislocation, thromboembolic event, and prosthetic joint infection (PJI) are the most common complications (3-5). PJIs have multifactorial causes, and extensive research has explored the risk factors for infection (3). Those associated with PJIs are both endogenous factors such as male gender, obesity, poor diabetic control, and active smoking (6, 7), and exogenous factors such as timing of prophylactic antibiotics, surgical site preparation, and laminar airflow (8-10). PJI as reason for reoperation has gradually increased in recent decades and is now the most common indication for reoperation within two years of primary THA (4). PJIs are associated with increased mortality (11-13), lower QoL (13, 14), and higher costs for healthcare providers (15, 16). In an attempt to reduce the PJI burden, the Swedish Orthopedic Association and Swedish National Patient Insurance started 2009 a nationwide, multidisciplinary infection control program: PRISS (Prosthetic Related Infections Shall be Stopped). All orthopedic surgical units (n = 81) in Sweden participated in the PRISS project and were reviewed. The goal of the project was to reduce PJI incidence following total hip and knee arthroplasty in Sweden by 50%. A study by Lindgren et al (17) estimated the PJI incidence to 0.9% after primary THA, the years prior to PRISS, 2005-2008. The effect of the PRISS project on PJI after THA is however unknown. The primary aim of the present study was therefore to investigate if the PRISS project reduced the incidence of PJI following THA; the secondary aims were to evaluate other possible benefits of the initiative, i.e., shorter time to diagnosis, and to identify pathogens involved in PJIs.

For peer review only - http://bmjopen.bmj.com/site/about/guidelines.xhtml

For peer review only

Methods and analysis

Design

The PRISS study is a cohort study.

Registries

All public and private orthopedic clinics performing THA operations report to the Swedish Arthroplasty Register (SAR). Approximately 98% of all THAs in Sweden are reported to the registry (4). Details such as indication for surgery, type of implant, surgical approach, body mass index (BMI), and American Society of Anesthesiology class (ASA) are reported. The Swedish Prescribed Drug Registry (SPDR) is operated by the Swedish National Board of Health and Welfare, and all pharmacies in Sweden automatically report complete details regarding dispensed drugs, including type of drug, amount dispensed, instructions from prescribing doctor, and dates of prescription and dispensing.

Swedish National Patient Insurance and the PRISS project

The Swedish National Patient Insurance (SNPI) is a public insurance company jointly owned by its policy holders, the Swedish regions and county councils. Its primary goals are to insure its owners, handle claims, compensate patients affected by health-care-associated complications, and address patient safety. In 2008, 33% of all claims were from the orthopedic specialty (18). In 2009,the PRISS project was initiated jointly by SNPI and the Swedish Orthopedic Association (18). The project started in 2009 and by 2012 all orthopedic units (n = 81) had been enrolled in it. It was an interdisciplinary project for safer THA and TKA operations involving several professional organizations such as the Swedish associations for orthopedic, infectious disease specialists, surgical nursing, orthopedic nursing, physical therapy, and infection control. A self-assessment tool, site inspections, and expert group evaluations were performed on all units to evaluate the routines before, during and after

For peer review only - http://bmjopen.bmj.com/site/about/guidelines.xhtml

For peer review only

arthroplasty surgery (18). Eighty-five percent of all implemented measures were improvement in procedures and protocols. Factors such as optimizing patients before surgery, basic hygiene routines, optimizing antibiotic prophylaxis, improving operating theatres (e.g. CFU measurements, locked rooms and maximum number of persons in operating rooms), and postoperative wound care were among those factors addressed (Table 1).

Table 1. Examples of procedures and improvements addressed in the PRISS project, 2009–2012.

Pre-operative	Operation environment	Post-operative
No-smoking information, medicines	Improved hygiene routines, standardization of protocols	Wound treatment, protocol for changing dressing, sterile environment
BMI limit	CFU measurement (<5 CFU/m^3)	Early detection
Diabetic control	Locked operation room	Patient information, sign of infection, contact information
Hemoglobin levels	Maximum number of people in OR	
Patient information		Removing sutures at department
Skin-sanitizing procedures	Normothermia, regular check of body temperature	Early detection of infection, phone screening 1–2 weeks post-op
Full body examination	Improved and disposable clothing for all personnel	Follow-up instructions for primary health care
Clear criteria for cancellation, active infection	WHO checklist for follow-up improvement	
Preoperative decolonization	Antibiotic prophylaxis, type, timing, dose and intervals	

BMI, body mass index; CFU, colony forming units; WHO, World Health Organization.

For peer review only

For peer review only - http://bmjopen.bmj.com/site/about/guidelines.xhtml

However, the impact of optimization of these measures in the setting of the PRISS project, on the PJI risk, has not been explored.

Study design, participants, and setting

A new method of postoperative infection surveillance introduced by Lindgren et al. combined national medical registry data with medical record review (17), determining PJI cumulative incidence within 2 years of primary THA to be 0.9% (95% CI 0.9-1.0) between 2005 and 2008. From that study, we extracted patient demographic data, on patients with a primary THA (all indications) and a PJI diagnosis to compare with the post PRISS cohort (Table 2).

Table 2. Demographic data for patients undergoing primary total hip arthroplasty, pre (2005–2008) and post PRISS (2012–2014).

	**Pre PRISS** (*n* = 49,259)	**Post PRISS** (*n* = 49,946)
Mean age (SD)	69 (11)	69 (11)
Female, sex	50 (29,078/49,259)	50 (28,645/49,946)
BMI ≥ 30	22 (2612/11,969)	24 (11,227/47,445)
ASA classification ≥ 3	17 (2252/13,069)	20 (9595/48,868)
Indication for operation		
Primary OA	83 (40,884/49,207)	80 (39,632/49,804)
Secondary OA	0 (22/49,207)	2 (1183/49,804)
Acute trauma, hip fracture	7 (3348/49,207)	9 (4325/49,804)
Complication trauma	3 (1413/49,207)	3 (1715/49,804)
Sequelae of childhood hip disease	2(1001/49,207)	2 (936/49,804)
Femoral head necrosis	3 (1235/49,207)	2 (1162/49,804)
Inflammatory joint disease	2 (1016/49,207)	1 (562/49,804)
Tumor	1 (288/49,207)	1 (291/49,804)
Surgical approach		
Direct lateral	44 (21,317/48,889)	48 (23,836/49,942)
Posterior	55 (26,786/48,889)	51 (25,654/49,942)
Other[1]	2 (786/ 48,889)	1 (454/49,942)
Implant fixation, any cemented[2]	89 (43,376/48,761)	82 (40,709/49,945)

Data shown as % (*n*) unless otherwise indicated.
BMI, body mass index; ASA American Society of Anesthesiology class, OA, osteoarthritis; PJI, prosthetic joint infection; MIS, minimally invasive surgery; SD, standard deviation.
[1] Trochanteric osteotomy, minimally invasive surgery.
[2] Cemented, hybrid, and reversed hybrid.

For peer review only

For peer review only - http://bmjopen.bmj.com/site/about/guidelines.xhtml

The present study used the same method, including diagnostic criteria for PJI (19), to determine the incidence of PJI within 2 years of primary THA over the 2012–2014 period. From SAR we extracted all primary THA procedures (*n* = 49,946 in 45,723 patients) between 1 January 2012 and 31 December 2014 (Figure 1). Using personal identity numbers, we matched this cohort with the SPDR and identified 2240 patients undergoing a total of 2569 primary THAs with continuous outpatient consumption of antibiotics for a minimum of 4 weeks minimum of 2 years after each primary THA. For each THA, a questionnaire was sent to the operating unit, where a physician reviewed the medical records of that patient to verify that he/she had been treated for PJI. From the questionnaire, information on date of diagnosis, clinical presentation, and surgical treatment was obtained (Supplemental Table 1). Ninety-nine percent (*n* = 2531/2569) of the questionnaires were returned along with the microbiology results. PJI after revision THA as well as superficial infections were excluded.

Patient and public involvement

None.

Statistics

Continuous variables were presented as means (with standard deviations) and medians (with inter-quantile ranges), as appropriate. The χ^2 test and Fisher's exact test were used to compare categorical data such as sex, BMI ≥ 30, and ASA ≥ 3. The *t*-test was used to evaluate between-group differences in age. Cumulative incidence of PJI with 95 % confidence intervals were estimated using the Kaplan-Meier estimator. Hazard ratios with 95 % confidence intervals were estimated using Cox regression adjusted for age, sex, and diagnosis (OA/other). Patients were followed for two years from primary operation until the first PJI and censored for death and revision for other causes than PJI. A sensitivity analysis, in the

For peer review only

For peer review only - http://bmjopen.bmj.com/site/about/guidelines.xhtml

part of the cohort with complete data, a Cox regression also adjusted for ASA and BMI were estimated.

All tests were two-tailed, and statistical significance was defined as a *p*-value < 0.05 or a 95% confidence interval (CI)) excluding an odds ratio (OR) equal to 1.00. Statistical analyses were performed using SPSS version 25 (IBM Corp., Armonk, NY, USA).

Results

Five hundred and eighty patients treated for a PJI were identified (Supplemental Table 2), resulting in a nationwide 2-year cumulative incidence of 1.2% [95% CI 1.1-1.3] over the 2012–2014 period as compared to 0.9% [95% CI 0.8-1.0] in the 2005-2008 period. There was an increasing trend of PJI incidence during the study period (Figure 2) and a significant increase of 30% during for the whole study period p<0.001. However, the multivariate Cox regression model indicated no statistically significant difference in the cumulative incidence pre- versus post-PRISS PJI (HR 1.1 [95% CI 0.9–1.3],).

The highest incidence was noted in university hospitals (1.5%), followed by regional (1.4%) and county and private hospitals (both 0.9%). After controlling for the potential confounders of age, sex, indication for surgery, BMI ≥ 30, and ASA ≥ 3, we found no statistically significant differences in PJI incidence among the different hospital classes. For individual orthopedic units, the cumulative incidence ranged from 0.0 to 5.9%; in two units the incidence had decreased and in three it had increased compared with before the PRISS project. The median time from primary THA to PJI diagnosis was 23 days (IQR 48) (Supplemental figure 1), not significantly different from the 24 days before the PRISS project (IQR 46, *p*=0.51).

In Table 3 the distribution of the microbial agents isolated from PJIs pre and post PRISS is presented.

For peer review only - http://bmjopen.bmj.com/site/about/guidelines.xhtml

Table 3. Distribution of microorganisms isolated from prosthetic joint infections of the hip, pre (2005-2008) and post the PRISS project (2012-2014)

Pathogens	Pre PRISS (n=442)	Post PRISS (=580)
Streptococcus spp.	7% (32)	8% (44)
Staphylococcus spp.		
Staphylococcus aureus	20% (89)	26% (150[1])
Coagulase-negative staphylococci	24% (108)	21% (122)
Enterococcus spp.	4% (16)	3% (20)
Enterobacterales	3% (15)	4% (25)
Anaerobes	1% (3)	3% (17)
Miscellaneous[2]	3% (13)	1% (6)
Polymicrobial[3]	27% (120)	25% (146)
Unknown[4]	10% (46)	9% (50)

[1] Including one methicillin-resistant *Staphylococcus aureus*.
[2] e.g. *Bacillus, Corynebacterium, Mycobacterium, Pseudomonas.*
[3] Polymicrobial findings (2–4 organisms). Pre PRISS 100% of polymicrobial PJIs included *Staphylococcus spp*. vs. 82% post PRISS project.
[4] Culture-negative PJIs and missing data.

The most common microorganisms, isolated in pure culture or as part of polymicrobial growth, were *Staphylococcus* spp. (72% pre vs 68% post, *p*=0.9). In pure culture, *Staphylococcus aureus*, was the most abundant species, with a minor increase post PRISS (20% vs 26%, *p*=0.04), and one isolate was found to be methicillin resistant (i.e., MRSA) vs. none pre PRISS. Coagulase-negative staphylococci (CoNS) were the second most commonly isolated group of bacteria (21% vs 24%, *p*=0.2). The number of polymicrobial PJIs, with 2–4 different bacterial species, did not differ significantly pre vs post PRISS (27% vs 25%, *p*=0.5).

Discussion

We investigated the effect of PRISS on PJI incidence in patients with primary THA in the years following the project (i.e., 2012–2014) with a follow-up period of 2 years. We also examined time to diagnosis and infecting microorganisms. We found a 30 % increase in PJI incidence after as compared to before the PRISS project, i.e. 1.2 vs. 0.9% (17), but there was no statistically significant difference, after controlling for confounders such as BMI, ASA,

For peer review only - http://bmjopen.bmj.com/site/about/guidelines.xhtml

and indication for surgery. Time to diagnosis remained unchanged pre vs. post PRISS (i.e., median 23 vs. 24 days).

Different sources of error may have affected the PJI incidence level reported here. One source of error was that the specific reason for antibiotic prescription could not be established in all cases, with a risk of selection bias For example, if a patient had moved from the county where s/he initially underwent surgery, local medical record review was impossible. Another limitation concerns identifying the patient cohort through SPDR. Since SPDR only handles outpatient prescriptions, patients who died in hospital or were hospitalized during the course of treatment could not be detected; with a risk of introducing immortal time bias. However the mortality rate is very low following arthroplasty (20), limiting this effect. According to the Swedish national guidelines (21), the advocated antibiotic treatment for PJI is 2 weeks of intravenous and 10 weeks of oral antibiotic therapy. Inpatient care for such long time is rare in Sweden, which also minimizes the immortal time bias. In addition, the sensitivity of the 2011 MSIS criteria has been questioned (22). All these limitations may serve to underestimate the true incidence of PJI. Nevertheless, the study was performed using the exact same methodology as in the prior study by Lindgren et al, (17) establishing the PJI incidence before PRISS and having the same limitations in both studies making the results comparable. The study was non-randomized thus limiting its power to establish causality. The time-period for the post PRISS cohort was selected to be immediately following the completion of the PRISS project, minimizing the regression toward the mean effect. SAR started to collect BMI and ASA data in 2008, limiting the number of patients for whom this information is available. Over the study period we could identify a trend of increasing comorbidity of the patients (age, BMI, ASA class) and proportion of patients with hip fracture/secondary OA/sequelae after fracture.

For peer review only

As the cause of PJI is multifactorial any initiative to reduce the infection incidence must be broad and include all health care disciplines involved. The PRISS project included implementations of a bundle of actions to improve pre-, intra- and post-operative care following expert reviews(18). All recommendations were voluntary for the orthopaedic units, possibly limiting the effect of best-practice methods. It is also possible that there are unknown confounders contribution to an increase in PJI incidence that were not measured.

Most infections were diagnosed in the first two weeks following THA and infections were only sporadically identified after the first 90 days (Supplemental figure 1). This implies that most infections originate from the surgical procedure or during the early postoperative period, indicating that properly timing the first dose of prophylactic antibiotics as well as very careful wound care in the early postoperative period are essential. This also implies that the improved post-operative procedures and protocols of PRISS, such as wound treatment protocols, phone screening, and patient information, had limited impact on PJI incidence. There was no change in time to diagnosis compared with before the PRISS project (i.e., 23 vs. 24 days). This time to diagnosis is likely acceptable, as infection treatment with debridement, antibiotics, and implant retention (DAIR) has a chance of successful outcome (i.e., infection eradication) if initiated within 28 days of infection for acute infections (21, 23-25). If a PJI has progressed for more than 4 weeks extensive surgery, i.e. one or two stage exchange is advocated for a successful outcome.

The risk of PJI following the first year of surgery is approximately 1% annually (3, 17), a risk that seemed to be unaffected by the PRISS project. Late infections include both those contracted during surgery or the early postoperative phase progressing to chronic PJIs, and

For peer review only - http://bmjopen.bmj.com/site/about/guidelines.xhtml

acute hematogenous infections, which by definition could not be affected by the PRISS project measures. Although hematogenous infections can be treated successfully with a DAIR procedure within 3 weeks of infection onset (25), this treatment algorithm is less likely to be successful in a procedure for chronic PJI (24, 26, 27). In this study we have not tried to distinguish between the two types of late infections.

The PJI incidence before PRISS was lower at the beginning of the study period, and there was a trend of increasing incidence at the end of 2008(17). A trend of increasing PJI incidence could also be detected over the 2012–2014 period, representing a continuation of the increasing incidence noted later in the 2007–2008 period (Figure 2). This rising trend could have been because determination to achieve improvement diminished following the end of PRISS. The PRISS project was a national intervention with broad support from participants from all professional organizations in collaboration with SNPI and all orthopedic units. Despite broad support and an evidence-based approach, PRISS project did not reduce PJI incidence. During the 2008–2014 period, the number of patients with severe systemic disease (ASA $\geq$ 3) increased, possibly contributing to the increasing PJI incidence over this period(28). More obese patients and patients with hip fractures underwent THA post PRISS (Table 2). Early operation is very important for a patient with a hip fracture (29) , but may also risk non-compliance to preoperative routines, thus contributing to the PJI risk. Thompson et al concluded that the PRISS project did not had any effect on PJI incidence after primary total knee arthroplasty (TKA) with similar pre and post PJI incidence(30), but did not explore the effect of co-morbidities . If the trend of increasing co-morbidities apply to the patients with TKA, this could imply that PRISS project could have had an effect on the PJI incidence for TKA (30) . However, fracture as indication for primary TKA is rare as compared to THA, limiting this effect on PJI incidence for TKA. Although PJI incidence has progressively

For peer review only - http://bmjopen.bmj.com/site/about/guidelines.xhtml

increased over the years in Sweden, it is comparable to that reported in an international context (31, 32). Some risk factors for infection cannot be influenced (e.g., sex, age, and reason for THA), whereas some, although modifiable (e.g., heart failure, diabetes, and obesity), are hard to influence especially if the patient is in pain and has difficulty walking before THA, meaning that they were largely unaffected by the preoperative measures of the PRISS initiative. However, it is possible that the PJI incidence might have been even higher without the PRISS project and its various benefits, such as more patients receiving correct initial treatment and therefore being cured of infection (3, 30).

Staphylococci were by far the most common etiological agent of PJI, in agreement with international reports (33) and national observations before PRISS (17, 34). Since only one methicillin-resistant *S. aureus* (MRSA) case was found, the vast majority of *S. aureus* were susceptible to most antibiotics. However, on the contrary, many of the CoNS were not determined to species level, and *S. epidermidis*, the most common staphylococcal species among CoNS causing PJI, are in contrast to *S. aureus* often multidrug resistant (35). Whether a pattern of increasing multidrug resistance could explain the increasing PJI incidence was not evaluated here.

Conclusions

The PRISS project did not reduce the PJI incidence and the time to diagnosis remained unchanged. This study indicates a lack of impact of non-mandatory recommended measures, despite being evidence based, to be implemented to reduce multifactorial complications such as PJI of the hip when the incidence is low. Higher proportions of obese, comorbid, and fracture patients likely explain the minor increase in PJI incidence, and it is possible that in modern health care, endogenous risk factors might affect the PJI incidence more than exogenous (and modifiable) risk factors. Further studies of patients with a high risk of PJI,

For peer review only - http://bmjopen.bmj.com/site/about/guidelines.xhtml

such as fracture, revision surgery and comorbid patients, are warranted to reduce overall incidence of PJI and its severe complications. An increased focus on PJIs may have contributed to a better treatment outcome, but this needs further studies.

Ethical approval

This study was approved by the Regional Ethical Review Board of Gothenburg, Sweden (reference number 2017/329-17).

Conflicts of interests

The authors declare they have no conflicts of interests.

Author contributions

PW, OR, PWR, MG, BS and VL designed the study. VL had project supervision. PW, MG and JN analyzed the data. PW wrote the manuscript with contributions from OR, PWR, JN, MG, BS and VL. All authors approved the final version of the manuscript.

Acknowledgments

The authors would like to thank all orthopedic units and SAR contact physicians for review of the medical records. The authors would also like to thank Ann-Britt Wall, Department of Orthopedics Region Örebro County, for administrative support.

Funding

The authors would like to thank the funding organizations, Research Committee of Örebro University, Region Örebro County (Grant no: OLL-917121), Sweden and Patientförsäkringen LÖF, Sweden (Grant no:NA), for financial support of the study. The funders had no role in study design, data collection and analysis, decision to publish or preparation of the manuscript.

For peer review only

For peer review only - http://bmjopen.bmj.com/site/about/guidelines.xhtml

Data sharing

All data relevant to the study are included in the article or uploaded as supplementary information.

Figures captions

Figure 1. Study flowchart of primary THR with antibiotic treatment ≥28 days followed up for review of medical records.

Figure 2. Trend of cumulative prosthetic joint infection incidence pre and post PRISS, 2005-2014.

Supplemental Figure 1. Time from total hip arthroplasty to prosthetic joint infection diagnosis following the PRISS project, 2012–2014. Each bar represents 1 week.

For peer review only

References

1. Ritchie H, Roser M. Age Structure. 2019 [cited 2021 04/20]; Available from: https://ourworldindata.org/age-structure.
2. Learmonth ID, Young C, Rorabeck C. The operation of the century: total hip replacement. Lancet. 2007 Oct 27;370(9597):1508-19.
3. Tande AJ, Patel R. Prosthetic joint infection. Clin Microbiol Rev. 2014 Apr;27(2):302-45.
4. Kärrholm J, Rogmark C, Nauclér E, Vinblad J, Mohaddes M, Rolfson O. Swedish Hip Arthroplasty Register Annual Report 2018. . 2018;2018(1st January).
5. Healy WL, Iorio R, Clair AJ, Pellegrini VD, Della Valle CJ, Berend KR. Complications of Total Hip Arthroplasty: Standardized List, Definitions, and Stratification Developed by The Hip Society. Clin Orthop Relat Res. 2016 Feb;474(2):357-64.
6. Iannotti F, Prati P, Fidanza A, Iorio R, Ferretti A, Pèrez Prieto D, et al. Prevention of Periprosthetic Joint Infection (PJI): A Clinical Practice Protocol in High-Risk Patients. Trop Med Infect Dis. 2020 Dec 11;5(4).
7. Sørensen LT. Wound healing and infection in surgery. The clinical impact of smoking and smoking cessation: a systematic review and meta-analysis. Arch Surg. 2012 Apr;147(4):373-83.

For peer review only - http://bmjopen.bmj.com/site/about/guidelines.xhtml

8. Alamanda VK, Springer BD. The prevention of infection: 12 modifiable risk factors. Bone Joint J. 2019 Jan;101-b(1_Supple_A):3-9.

9. Lenguerrand E, Whitehouse MR, Beswick AD, Kunutsor SK, Burston B, Porter M, et al. Risk factors associated with revision for prosthetic joint infection after hip replacement: a prospective observational cohort study. Lancet Infect Dis. 2018 Sep;18(9):1004-14.

10. Dale H, Fenstad AM, Hallan G, Havelin LI, Furnes O, Overgaard S, et al. Increasing risk of prosthetic joint infection after total hip arthroplasty. Acta Orthop. 2012 Oct;83(5):449-58.

11. Natsuhara KM, Shelton TJ, Meehan JP, Lum ZC. Mortality during total hip periprosthetic joint infection. J Arthroplasty. 2019 Jul;34(7s):S337-s42.

12. Gundtoft PH, Pedersen AB, Varnum C, Overgaard S. Increased Mortality After Prosthetic Joint Infection in Primary THA. Clin Orthop Relat Res. 2017 Nov;475(11):2623-31.

13. Wildeman P, Rolfson O, Söderquist B, Wretenberg P, Lindgren V. What Are the Long-term Outcomes of Mortality, Quality of Life, and Hip Function after Prosthetic Joint Infection of the Hip? A 10-year Follow-up from Sweden. Clin Orthop Relat Res. 2021 May 31.

14. Poulsen NR, Mechlenburg I, Soballe K, Lange J. Patient-reported quality of life and hip function after 2-stage revision of

For peer review only - http://bmjopen.bmj.com/site/about/guidelines.xhtml

chronic periprosthetic hip joint infection: a cross-sectional study. Hip Int. 2018 Jul;28(4):407-14.

15. Peel TN, Cheng AC, Lorenzo YP, Kong DC, Buising KL, Choong PF. Factors influencing the cost of prosthetic joint infection treatment. J Hosp Infect. 2013 Nov;85(3):213-9.

16. Lora-Tamayo J, Senneville É, Ribera A, Bernard L, Dupon M, Zeller V, et al. The Not-So-Good Prognosis of Streptococcal Periprosthetic Joint Infection Managed by Implant Retention: The Results of a Large Multicenter Study. Clin Infect Dis. 2017 Jun 15;64(12):1742-52.

17. Lindgren V, Gordon M, Wretenberg P, Karrholm J, Garellick G. Deep infection after total hip replacement: a method for national incidence surveillance. Infect Control Hosp Epidemiol. 2014 Dec;35(12):1491-6.

18. Gustafson P, Schultz T, Stefansdottir A. PRISS – ProsthesisRelated Infections Shall be Stopped 2014.

19. Parvizi J, Zmistowski B, Berbari EF, Bauer TW, Springer BD, Della Valle CJ, et al. New definition for periprosthetic joint infection: from the Workgroup of the Musculoskeletal Infection Society. Clin Orthop Relat Res. 2011 Nov;469(11):2992-4.

20. Dale H, Børsheim S, Kristensen TB, Fenstad AM, Gjertsen JE, Hallan G, et al. Perioperative, short-, and long-term mortality related to fixation in primary total hip arthroplasty: a study on 79,557 patients in the -Norwegian Arthroplasty Register. Acta Orthop. 2020 Apr;91(2):152-8.

For peer review only - http://bmjopen.bmj.com/site/about/guidelines.xhtml

21. Swedish Society for Infectious Diseases. Vårdprogram för Led- och skelettinfektioner 2018. https://infektion.net/2018; 128]. Available from: https://infektion.net/wp-content/uploads/2018/11/2018-vardprogram-led-och-skelettinfektioner-final-2018-11-29.pdf.

22. Koh IJ, Cho WS, Choi NY, Parvizi J, Kim TK. How accurate are orthopedic surgeons in diagnosing periprosthetic joint infection after total knee arthroplasty?: A multicenter study. Knee. 2015 Jun;22(3):180-5.

23. Osmon DR, Berbari EF, Berendt AR, Lew D, Zimmerli W, Steckelberg JM, et al. Executive summary: diagnosis and management of prosthetic joint infection: clinical practice guidelines by the Infectious Diseases Society of America. Clin Infect Dis. 2013 Jan;56(1):1-10.

24. Zimmerli W. Clinical presentation and treatment of orthopaedic implant-associated infection. J Intern Med. 2014 Aug;276(2):111-9.

25. Wouthuyzen-Bakker M, Sebillotte M, Lomas J, Taylor A, Palomares EB, Murillo O, et al. Clinical outcome and risk factors for failure in late acute prosthetic joint infections treated with debridement and implant retention. J Infect. 2019 Jan;78(1):40-7.

26. Wouthuyzen-Bakker M, Sebillotte M, Lomas J, Kendrick B, Palomares EB, Murillo O, et al. Timing of implant-removal in

For peer review only - http://bmjopen.bmj.com/site/about/guidelines.xhtml

late acute periprosthetic joint infection: A multicenter observational study. J Infect. 2019 Sep;79(3):199-205.

27. Maillet M, Pavese P, Bruley D, Seigneurin A, François P. Is prosthesis retention effective for chronic infections in hip arthroplasties? A systematic literature review. Eur J Clin Microbiol Infect Dis. 2015 Aug;34(8):1495-502.

28. Kärrholm J, Lindahl H, Malchau H, Mohaddes M, Rogmark C, Rolfson O. The Swedish Hip Arthroplasty Register Annual Report 2015. 2016.

29. Simunovic N, Devereaux Pj Fau - Sprague S, Sprague S Fau - Guyatt GH, Guyatt Gh Fau - Schemitsch E, Schemitsch E Fau - Debeer J, Debeer J Fau - Bhandari M, et al. Effect of early surgery after hip fracture on mortality and complications: systematic review and meta-analysis. (1488-2329 (Electronic)).

30. Thompson O, A WD, Lindgren V, Gordon M, Robertsson O, Stefánsdóttir A. Similar periprosthetic joint infection rates after and before a national infection control program: a study of 45,438 primary total knee arthroplasties. Acta Orthop. 2022 Jan 3;93:3-10.

31. Gundtoft PH, Pedersen AB, Schønheyder HC, Møller JK, Overgaard S. One-year incidence of prosthetic joint infection in total hip arthroplasty: a cohort study with linkage of the Danish Hip Arthroplasty Register and Danish Microbiology Databases. (1522-9653 (Electronic)).

For peer review only - http://bmjopen.bmj.com/site/about/guidelines.xhtml

32. McMaster Arthroplasty Collaborative (MAC). Risk Factors for Periprosthetic Joint Infection Following Primary Total Hip Arthroplasty: A 15-Year, Population-Based Cohort Study. J Bone Joint Surg Am. 2020;2020;102(6):503-509(1535-1386 (Electronic)).

33. Flurin L, Greenwood-Quaintance KE, Patel R. Microbiology of polymicrobial prosthetic joint infection. Diagn Microbiol Infect Dis. 2019 Jul;94(3):255-9.

34. Stefánsdóttir A, Johansson D, Knutson K, Lidgren L, Robertsson O. Microbiology of the infected knee arthroplasty: report from the Swedish Knee Arthroplasty Register on 426 surgically revised cases. Scand J Infect Dis. 2009;41(11-12):831-40.

35. Månsson E, Bech Johannesen T, Nilsdotter-Augustinsson Å, Söderquist B, Stegger M. Comparative genomics of Staphylococcus epidermidis from prosthetic-joint infections and nares highlights genetic traits associated with antimicrobial resistance, not virulence. Microbial genomics. 2021 Feb;7(2).

For peer review only - http://bmjopen.bmj.com/site/about/guidelines.xhtml

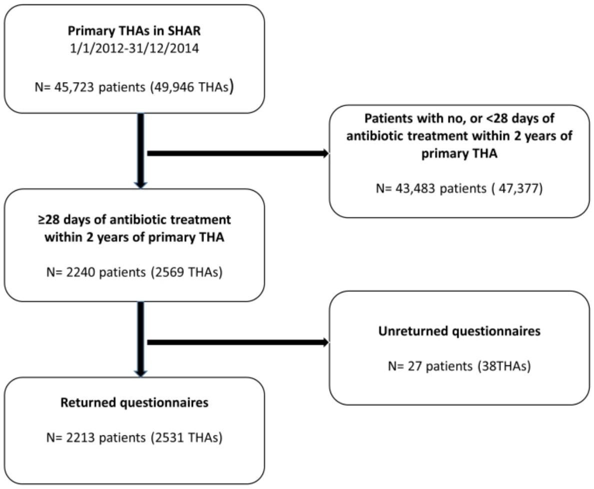

Figure 1. Study flowchart of primary THR with antibiotic treatment ≥28 days followed up for review of medical records.

338x190mm (300 x 300 DPI)

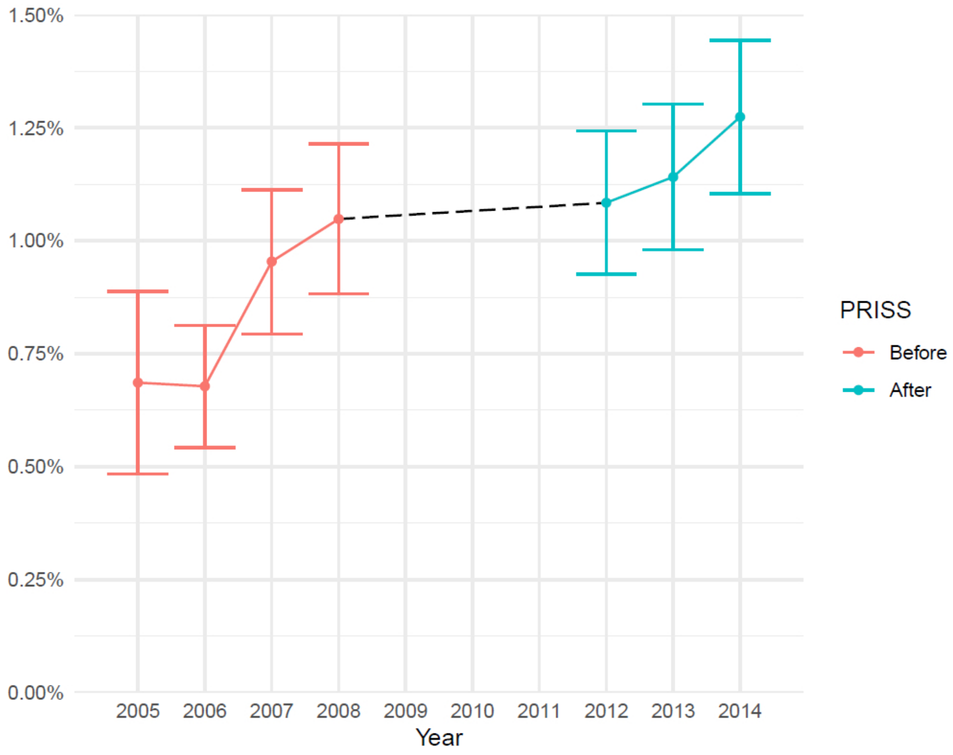

Figure 2. Trend of cumulative prosthetic joint infection incidence pre and post PRISS, 2005-2014.

88x66mm (300 x 300 DPI)

Supplemental Table 1. Diagnostic criteria walkthrough for patients treated for PJI pre PRISS (2005-08) post PRISS (2012-14); diagnostic criteria were verified sequentially.

Diagnostic criteria	(n=442)	(n=580)
1. Sinus tract communicating with the prosthesis	14 (64)	29 (168)
2. ≥2 positive periprosthetic cultures with same pathogen	59 (262)	53 (310)
3a + 3b. Presence of purulence in joint + elevated CRP/SR	5 (23)	5 (30)
3a + 3c. Presence of purulence + isolation of one periprosthetic pathogen in tissue or fluid	4 (19)	5 (31)
3b + 3c. Elevated CRP/SR + isolation of one periprosthetic pathogen in tissue or fluid	7 (29)	2 (10)
Clinical diagnosis or lack of information in questionnaires	10 (45)	5 (31)

Data presented as % (n)

CRP, C-reactive protein; SR, sedimentation rate.

Supplemental Table 2. Characteristics of patients with and without prosthetic joint infection (PJI) within 2 years of primary total hip placement surgery, 2012–2014.

	**PJI** (*n* = 580)	**Control** (*n* = 49,366)	***p*–value**
Mean age (SD)	71 (10)	69 (10)	<0.001
Female, sex	41 (240)	58 (28,405)	<0.001
BMI ≥ 30	33 (178)	24 (11,049)	<0.001
ASA classification ≥ 3	35 (200)	19 (9395)	<0.001
Indication for operation			<0.001
Primary OA	70 (401)	80 (39,231)	
Secondary OA	4 (22)	2 (1161)	
Acute trauma	12 (70)	9 (4255)	
Complication trauma	7 (40)	3 (1675)	
Sequelae of childhood hip disease	1 (7)	2 (929)	
Femoral head necrosis	3 (18)	2 (1144)	
Inflammatory joint disease	1 (13)	1 (547)	
Tumor	1 (6)	1 (285)	
Surgical approach			0.37
Direct lateral	51 (295)	48 (23,541)	
Posterior	48 (280)	51 (25,374)	
Trochanteric Osteotomy	0 (0)	0 (70)	
MIS[1]	1 (4)	1 (365)	
Implant fixation			0.22
Cemented	69 (402)	66 (32,530)	
Hybrid/reversed-hybrid	14 (80)	19 (9138)	
Uncemented	17 (98)	19 (9138)	

Data shown as % (*n*) unless otherwise indicated.

BMI, body mass index; ASA American Society of Anesthesiology class, OA, osteoarthritis; PJI, prosthetic joint infection; MIS, minimally invasive surgery; SD, standard deviation.

[1] MIS anterior, MIS posterior, Watson-Jones.

For peer review only

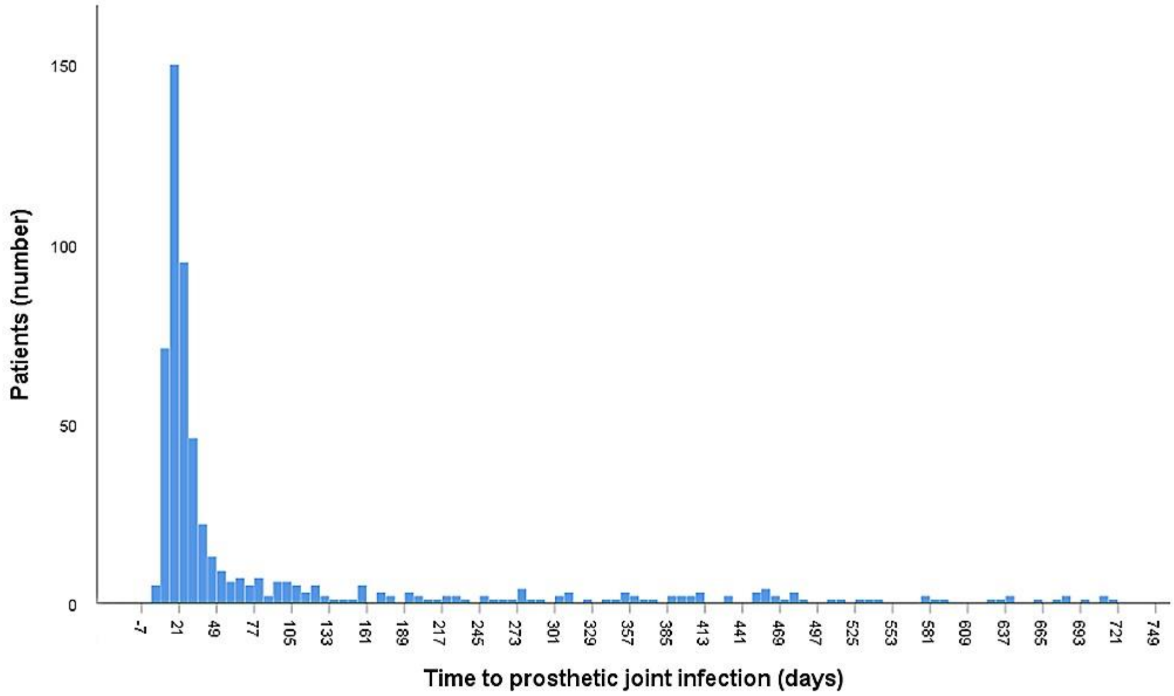

Supplemental Figure 1. Time from total hip arthroplasty to prosthetic joint infection diagnosis following the PRISS project, 2012–2014. Each bar represents 1 week.

STROBE Statement

Checklist of items that should be included in reports of observational studies

Section/Topic	Item No	Recommendation	Reported on Page No
**Title and abstract**	1	(*a*) Indicate the study's design with a commonly used term in the title or the abstract	1
		(*b*) Provide in the abstract an informative and balanced summary of what was done and what was found	3-4
**Introduction**			
Background/rationale	2	Explain the scientific background and rationale for the investigation being reported	4-5
Objectives	3	State specific objectives, including any prespecified hypotheses	5
**Methods**			
Study design	4	Present key elements of study design early in the paper	5
Setting	5	Describe the setting, locations, and relevant dates, including periods of recruitment, exposure, follow-up, and data collection	5-6
Participants	6	(*a*) *Cohort study*—Give the eligibility criteria, and the sources and methods of selection of participants. Describe methods of follow-up<br>*Case-control study*—Give the eligibility criteria, and the sources and methods of case ascertainment and control selection. Give the rationale for the choice of cases and controls<br>*Cross-sectional study*—Give the eligibility criteria, and the sources and methods of selection of participants	5-6
		(*b*) *Cohort study*—For matched studies, give matching criteria and number of exposed and unexposed<br>*Case-control study*—For matched studies, give matching criteria and the number of controls per case	
Variables	7	Clearly define all outcomes, exposures, predictors, potential confounders, and effect modifiers. Give diagnostic criteria, if applicable	5-7
Data sources/measurement	8*	For each variable of interest, give sources of data and details of methods of assessment (measurement). Describe comparability of assessment methods if there is more than one group	6,7
Bias	9	Describe any efforts to address potential sources of bias	9-11
Study size	10	Explain how the study size was arrived at	6,7
Quantitative variables	11	Explain how quantitative variables were handled in the analyses. If applicable, describe which groupings were chosen and why	7,8
Statistical methods	12	(*a*) Describe all statistical methods, including those used to control for confounding	7,8
		(*b*) Describe any methods used to examine subgroups and interactions	7,8
		(*c*) Explain how missing data were addressed	7,8
		(*d*) *Cohort study*—If applicable, explain how loss to follow-up was addressed<br>*Case-control study*—If applicable, explain how matching of cases and controls was addressed<br>*Cross-sectional study*—If applicable, describe analytical methods taking account of sampling strategy	7,8
		(*e*) Describe any sensitivity analyses	7,8

For peer review only - http://bmjopen.bmj.com/site/about/guidelines.xhtml

Section/Topic	Item No	Recommendation	Reported on Page No
**Results**			
Participants	13*	(a) Report numbers of individuals at each stage of study—eg numbers potentially eligible, examined for eligibility, confirmed eligible, included in the study, completing follow-up, and analysed	6,7
		(b) Give reasons for non-participation at each stage	
		(c) Consider use of a flow diagram	Figure 1
Descriptive data	14*	(a) Give characteristics of study participants (eg demographic, clinical, social) and information on exposures and potential confounders	Table 2
		(b) Indicate number of participants with missing data for each variable of interest	Figure 1
		(c) *Cohort study*—Summarise follow-up time (eg, average and total amount)	7
Outcome data	15*	*Cohort study*—Report numbers of outcome events or summary measures over time	8, Figure 2
		Case-control study—Report numbers in each exposure category, or summary measures of exposure	
		Cross-sectional study—Report numbers of outcome events or summary measures	
Main results	16	(*a*) Give unadjusted estimates and, if applicable, confounder-adjusted estimates and their precision (eg, 95% confidence interval). Make clear which confounders were adjusted for and why they were included	8
		(*b*) Report category boundaries when continuous variables were categorized	8
		(*c*) If relevant, consider translating estimates of relative risk into absolute risk for a meaningful time period	
Other analyses	17	Report other analyses done—eg analyses of subgroups and interactions, and sensitivity analyses	8,9
**Discussion**			
Key results	18		9
Limitations	19	Discuss limitations of the study, taking into account sources of potential bias or imprecision. Discuss both direction and magnitude of any potential bias	9-13
Interpretation	20	Give a cautious overall interpretation of results considering objectives, limitations, multiplicity of analyses, results from similar studies, and other relevant evidence	9-13
Generalisability	21	Discuss the generalisability (external validity) of the study results	13
**Other Information**			
Funding	22	Give the source of funding and the role of the funders for the present study and, if applicable, for the original study on which the present article is based	14

**Give information separately for cases and controls in case-control studies and, if applicable, for exposed and unexposed groups in cohort and cross-sectional studies.*

Note: An Explanation and Elaboration article discusses each checklist item and gives methodological background and published examples of transparent reporting. The STROBE checklist is best used in conjunction with this article (freely available on the Web sites of PLoS Medicine at http://www.plosmedicine.org/, Annals of Internal Medicine at http://www.annals.org/, and Epidemiology at http://www.epidem.com/). Information on the STROBE Initiative is available at www.strobe-statement.org.

For peer review only - http://bmjopen.bmj.com/site/about/guidelines.xhtml
